# Anti-Ulcerative Colitis Tanzawaic Acids from a Marine Algicolous Fungus, Penicillium steckii SCSIO 41040

**DOI:** 10.3390/md24050147

**Published:** 2026-04-22

**Authors:** Yingying Song, Jiamin Wang, Yuchen Lin, Jianglian She, Yalin Liu, Xiangxi Yi, Chenghai Gao, Junfeng Wang, Yonghong Liu

**Affiliations:** 1 Institute of Marine Drugs, Faculty of Pharmacy, Guangxi Key Laboratory of Marine Drugs, University Engineering Research Center of High-Efficient Utilization of Marine Traditional Chinese Medicine Resources, Guangxi, Guangxi University of Chinese Medicine, Nanning 530200, China; 16637610987@163.com (Y.S.); lyl20000627@163.com (Y.L.); yixiangxi2017@163.com (X.Y.); 2 Department of Pathophysiology, School of Medicine, Jinan University, Guangzhou 510632, China; wjm2275322055@163.com (J.W.); linyuchen9713@163.com (Y.L.); 3 Guangdong Key Laboratory of Marine Materia Medica/State Key Laboratory of Tropical Oceanography, South China Sea Institute of Oceanology, Chinese Academy of Sciences, Guangzhou 510301, China; shejianglian20@mails.ucas.ac.cn; 4 University of Chinese Academy of Sciences, 19 Yuquan Road, Beijing 100049, China

**Keywords:** *Penicillium steckii*, tanzawaic acids, intestinal barrier function, inflammation

## Abstract

Three new, previously undescribed tanzawaic acids, steckwaic acids H–J (**1**–**3**), and twenty-three known natural products (**4**–**26**) were isolated from the marine algicolous fungus *Penicillium steckii* SCSIO 41040. Structurally, compound **3** underwent a rare hydration reaction at the double bond of its carboxylic acid side chain. The chemical structures and stereochemistry were determined using comprehensive spectroscopic analyses, including NMR, electronic circular dichroism (ECD) calculations, and high-resolution electrospray ionization mass spectrometry (HRESIMS), and verified by literature comparison. The protective effect of tanzawaic acids on inflammatory damage to the intestinal epithelial barrier was assessed using an LPS-stimulated Caco-2/THP-1 co-culture model. Notably, immunofluorescence and Western blotting assays showed that compound **10** significantly enhanced the fluorescence signals and protein expression of ZO-1 and occludin, alleviated lipopolysaccharide (LPS)-induced intestinal barrier damage in Caco-2 cells, and contributed to the re-establishment of intestinal barrier homeostasis. Our findings demonstrate the critical role of tanzawaic acids in maintaining intestinal barrier integrity, identifying them as promising lead compounds for UC treatment.

## 1. Introduction

Tanzawaic acids are a class of polyketides characterized by a trans-decalin scaffold fused with a (2*E*, 4*E*)-penta-2,4-dienoic acid side chain [1]. Their structural diversity is further enriched by variable substituents, such as acrylic acid units, hydroxyl, acetoxyl, and methoxyl groups, and even halogen atoms, as well as distinct configurations of double bonds and chiral centers [2,3,4]. Since the first report in 1993, approximately one hundred tanzawaic acids have been isolated from various *Penicillium* species [5]. These compounds exhibit a wide range of biological activity, such as antimicrobial, cytotoxic, antimalarial, antitubercular, and anti-inflammatory effects [2,6,7,8,9,10,11]. Notably, tanzawaic acids have exhibited significant anti-inflammatory potential. Our earlier studies demonstrated that tanzawaic acids can suppress osteoclastogenesis in vitro and protect ovariectomized (OVX) mouse models against osteoporosis in vivo [5,11]. Moreover, several studies have demonstrated that tanzawaic acids can reduce the secretion of pro-inflammatory cytokines such as tumor necrosis factor-α (TNF-α) and interleukin-1β (IL-1β) [7]. These findings suggest that tanzawaic acids are promising lead compounds for the development of anti-inflammatory agents.

UC is a chronic inflammatory bowel disease characterized by persistent mucosal inflammation and oxidative stress [12,13]. Growing evidence suggests that intestinal barrier dysfunction plays a critical role in the pathogenesis and progression of UC [14]. The intestinal barrier consists of a network of endothelial cells, with intercellular interactions mediated by tight junctions (TJs), adhesion junctions (AJs), and gap junctions (GJs). In particular, TJs, composed of proteins such as occludin, claudins, zonula occludens (ZOs), and junctional adhesion molecules (JAMs), are essential for maintaining intestinal barrier integrity [15]. Although a number of pharmaceutical agents, such as antibiotics, 5-aminosalicylic acid (5-ASA) derivatives, corticosteroids, and immunosuppressants, are presently employed in clinical settings, these therapeutic modalities exhibit restricted efficacy and induce various adverse effects [16,17]. Hence, there is an urgent requirement for UC therapeutic drugs characterized by low costs, safety, and strong efficacy. Although tanzawaic acid derivatives have shown promising anti-inflammatory properties, their potential in the treatment of UC has not been explored.

**Figure 1:**
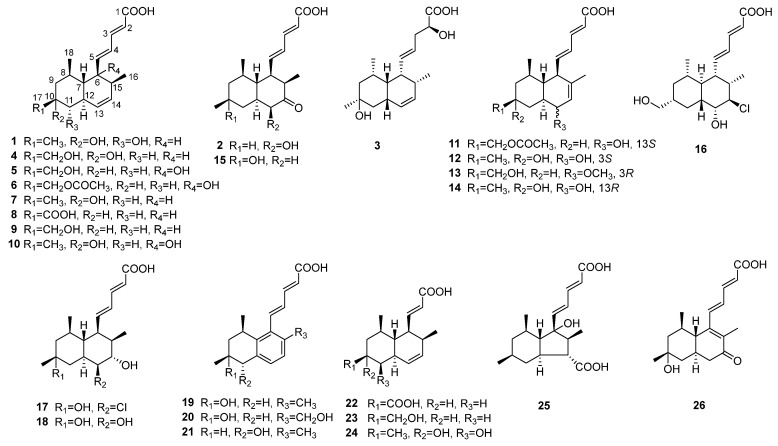
Chemical structures of compounds **1**–**26**.

During our ongoing research on tanzawaic acids form *Penicillium steckii* [5,11], a series of structurally diverse tanzawaic acids were identified (Figure 1), and their anti-ulcerative colitis effects were tested. Herein, we report the isolation, structural elucidation, and inflammatory activity of tanzawaic acids.

## 2. Results

**Figure 2:**
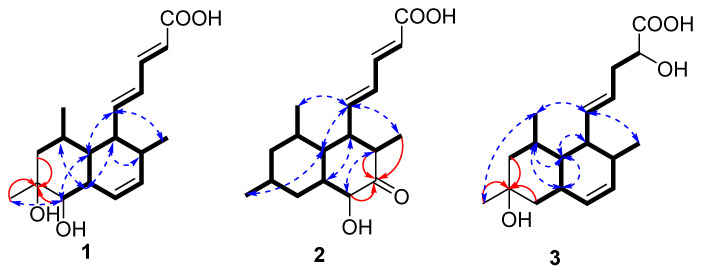
Key COSY (black bold lines), HMBC (red arrows), and NOESY (blue dotted line) correlations.

**Figure 3:**
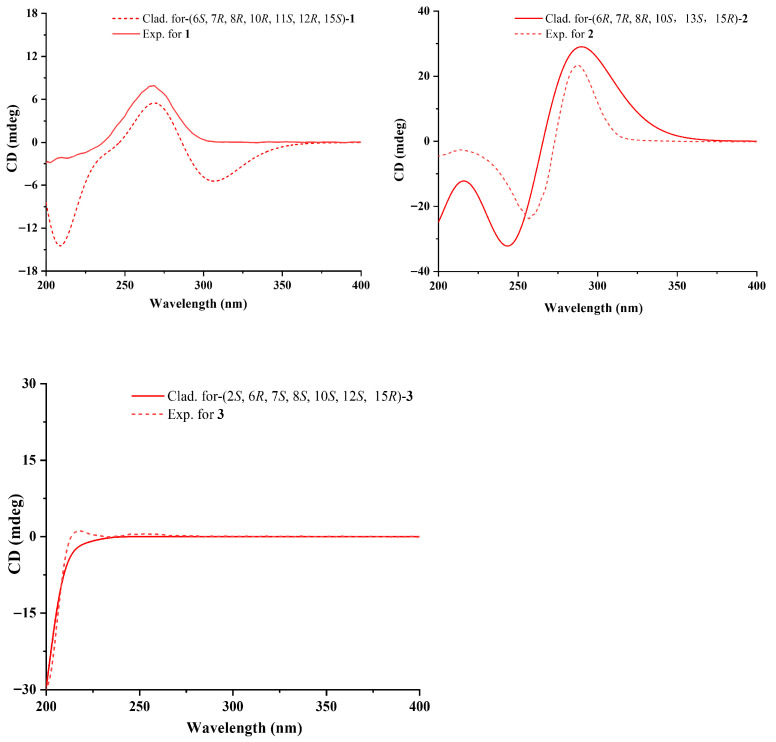
Experimental and calculated ECD spectra of compounds **1**–**3**.

Compound **1** was isolated as a yellow powder with the molecular formula of C_18_H_26_O_4_ based on the HRESIMS ions detected at *m*/*z* 305.1762 [M − H]^−^, which required six degrees of unsaturation. The ^1^H NMR and ^13^C NMR data (Table 1) indicated six olefinic methines (*δ*_C/H_ 122.0/5.76, CH-2; 143.4/7.10, CH-3; 126.8/6.17, CH-4; 148.3/6.17, CH-5; 129.3/5.93, CH-13; 132.2/5.59, CH-14), five methines (*δ*_C/H_ 48.8/2.37, CH-6; 44.6/0.95, CH-7; 30.9/1.62, CH-8; 43.3/2.07, CH-12; 36.1/2.09, CH-15), one methylene (*δ*_C/H_ 48.8/1.09, 1.54, CH_2_-9), three methyls (*δ*_C/H_ 16.3/0.90, CH_3_-16; 27.4/1.08, CH_3_-17; 21.8/0.81, CH_3_-18), one oxygenated methine (*δ*_C/H_ 76.4/2.81, CH-11), and two quaternary carbonyls (*δ*_C_ 168.5, C-1; 70.4, C-10). Six degrees of unsaturation could be accounted for by the presence of one carboxyl, six *sp^2^* carbon signals of three olefinic bonds, and two rings. The basic skeleton of the compound was deduced by COSY and HMBC data experiments (Figure 2). COSY correlations of H-6/H-7/H-8/H_2_-9, H-11/H-12/H-13/H-14/H-15, H-7/H-12 and HMBC correlations from H_2_-9 to C-10 and H-11 to C-10 revealed the presence of a decalin scaffold, which linked to three methyl groups at C-8, C-10, and C-15, respectively. The COSY data of H-2/H-3/H-4/H-5/H-6 pointed to a penta-2,4-dienoic acid chain connected at C-6, characteristic of tanzawaic acids. The remaining two hydroxyl groups were linked at C-10 and C-11, respectively, as determined by the HMBC signals of H_2_-9 to C-10 and H-11 to C-10. The planar structure was similar to those of tanzawaic acid E [18]. The relative configuration of compound **1** was determined by coupling constant data and NOESY correlations. The observed coupling constants (^3^*J*_H-2-H-3_ = 15.2 Hz, ^3^*J*_H-4-H-5_ = 15.0 Hz, and ^3^*J*_H-13-H-14_ = 9.6 Hz) indicated an *E*-configuration for the penta-2,4-dienoic acid side chain and a *Z*-configuration for the double bond in the ring. NOESY correlations (Figure 2) exhibited cross-peaks between H-12/H-6/H-8 and H-6/H-15 that indicated a co-facial arrangement, contrasting with those from H-5/H-7/H-11 on the opposite face. The absolute configuration of **1** was determined via the ECD spectrum (Figure 3) as 6*S*, 7*R*, 8*R*, 10*R*, 11*S*, 12*R*, 15*S*.

Compound **2** was obtained as a yellow oil and was assigned the molecular formula C_18_H_26_O_4_ via HRESIMS (*m*/*z* 305.1763 [M − H]^−^, calcd for C_18_H_25_O_4_^−^, 305.1758) and NMR data, with six degrees of unsaturation. Compound **2** and steckwaic acid J shared identical molecular formulas and functional groups, differing primarily in the hydroxyl group position [11]. Specifically, steckwaic acid J bore its hydroxyl group at C-10, whereas **2** carried this group at C-13 (*δ*_C_ 77.3), which was confirmed by COSY correlations of H-12 (*δ*_H_ 1.25)/H-13 (*δ*_H_ 3.88) and HMBC correlations from H-13 to C-14 (*δ*_C_ 210.4). The large coupling constants (15.4 Hz) of the double bonds *Δ*^2^ and *Δ*^4^ suggested their *E*-configuration. NOESY correlations (Figure 2) of H-7 (*δ*_H_ 1.40)/H_3_-17 (*δ*_H_ 0.87) and H-5 (*δ*_H_ 6.11)/H-7/H_3_-16 (*δ*_H_ 0.82)/H_3_-18 (*δ*_H_ 0.87) suggested the co-facial orientation of these groups, whereas correlations of H-12/H-6 (*δ*_H_ 1.99) and H-13/H-15 (*δ*_H_ 2.44) placed them on the opposite face. The experimental ECD spectra of **3** matched well with the calculated spectra for (6*R*, 7*R*, 8*R*, 10*S*, 12*S*, 13*S*, 15*R*)-**2**, confirming the absolute configurations of **2** as depicted.

Compound **3** was obtained as a yellow powder and has the molecular formula of C_18_H_28_O_4_ as established from HRESIMS analysis, indicative of five degrees of unsaturation. The NMR data of **3** were comparable with those of tanzawaic acid E [18], whereas it lacked one double-bond pair (*δ*_C_ 67.9 and *δ*_C_ 43.3). The loss of the double bond occurred in the penta-2,4-dienoic acid chain of compound **3**, where an unusual addition reaction introduced a water molecule. This phenomenon was confirmed by COSY correlations observable among H-2 (*δ*_H_ 4.24)/H_2_-3 (*δ*_H_ 2.19/2.20)/H-4 (*δ*_H_ 5.32). The ^3^*J*_H-4-H-5_ and ^3^*J*_H-13-H-14_ coupling constants were 15.7 Hz and 9.5 Hz, respectively, which indicated *E*- and *Z*-configurations for the two double bonds. The NOESY correlations of H-5 (*δ*_H_ 5.61)/H_3_-16 (*δ*_H_ 0.86)/H_3_-18 (*δ*_H_ 0.88) and H_3_-18/H_3_-17 (*δ*_H_ 1.07) suggested that these two protons were co-facial, whereas correlations of H-8 (*δ*_H_ 1.62)/H-7 (*δ*_H_ 0.78)/H-12 (*δ*_H_ 2.19) and H-7/H-6 (*δ*_H_ 2.22) placed them on the opposite face. Considering the limited contribution of the flexible side chain to the ECD spectrum, and the lack of NOESY signals of H-2, a truncated model was used for the ECD calculations. The ECD calculations of the truncated model matched well with the experimental curves (Figure 3), confirming the 6*R*, 7*S*, 8*S*, 10*S*, 12*S*, 15*R* absolute configuration for **3**. The absolute configuration at C-2 was determined by NMR calculations using the gauge-independent atomic orbital (GIAO) strategy at the B3LYP/6-31+G (d, p) level of theory with the PCM (DMSO-*d*_6_) solvation model [19,20]. As a result, the calculated NMR data, with a high DP4^+^ probability of 98.19%, indicated 2*S*-**4** to be the most probable configuration. Therefore, the absolute configuration of **3** was established as 2*S*, 6*R*, 7*S*, 8*S*, 10*S*, 12*S*, 15*R*.

The known metabolites isolated from *Penicillium steckii* SCSIO 41040 included tanzawaic acid O (**4**) [2], penitanzacid A (**5**) [1], penicisteck acid Q (**6**) [5], tanzawaic acid C (**7**) [3], tanzawaic acid F (**8**) [18], tanzawaic acid M (**9**) [2], tanzawaic acid V (**10**) [3], 18-*O*-acetyltanzawaic acid R (**11**) [21], tanzawaic acid Z1 (**12**) [22], 13*R*-tanzawaic acid S (**13**) [21], 10-hydroxytanzawaic acid Q (**14**) [22], steckwaic acid J (**15**) [11], penicisteck acid S (**16**) [5], penitanzacid E (**17**) [1], penicisteck acid R (**18**) [5], tanzawaic acid D (**19**) [23], tanzawaic acid G (**20**) [11], penicisteck acid I (**21**) [5], steckwaic acid F (**22**) [21], steckwaic acid G (**23**) [21], penitanzacid H (**24**) [1], penicisteck acid P (**25**) [5], and penitanzacid F (**26**) [1]. These compounds were identified as the principal metabolites of the fungus through comparison of their spectroscopic data with previously reported values.

**Figure 4:**
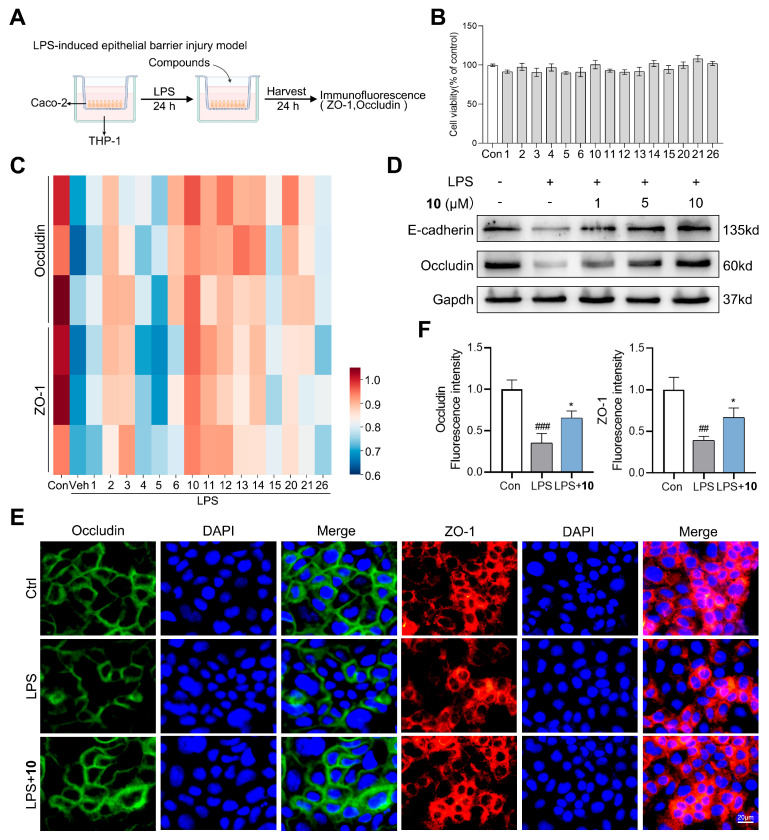
Effects of tanzawaic acids on the LPS-induced epithelial barrier injury model of Caco-2/THP-1 cells. (**A**) Schematic diagram of the Caco-2/THP-1 co-culture model for LPS-induced epithelial barrier injury. (**B**) Effects of tanzawaic acids on the cell viability of Caco-2 cells. (**C**) Occludin and ZO-1 expression in Caco-2 cells was determined by immunofluorescence of tanzawaic acids (10 μM). (**D**) Representative image of the Western blotting assay and the relative protein expression levels of E-cadherin and occludin in Caco-2 cells. (**E**) Occludin and ZO-1 expression in Caco-2 cells was determined by immunofluorescence of compound **10** (10 μM). (**F**) Quantitative analysis of (**E**). Values are expressed as mean ± SD. ^##^ *p* < 0.01, ^###^ *p* < 0.001 vs. control group; * *p* < 0.05 vs. LPS group.

Ulcerative colitis remains a major global health burden with limited therapeutic options. The Caco-2/THP-1 co-culture system models both intestinal epithelial barrier function and immune cell-mediated inflammation, closely recapitulating ulcerative colitis’ pathogenesis for anti-UC drug evaluation. In this study, we evaluated the anti-UC potential of the tanzawaic acids isolated from the marine fungus *Penicillium steckii* SCSIO 41040 using an in vitro LPS-stimulated Caco-2/THP-1 co-culture model (Figure 4A) [24,25]. Initial cytotoxicity screening in Caco-2 cells revealed that the compounds maintained >90% cell viability at 10 μM, and they were further selected for subsequent pharmacological assessment (Figure 4B). Proteins such as ZO-1, claudin-1, and occludin constitute the tight junction protein family, which is essential for maintaining intestinal barrier integrity [26,27]. Therefore, this study initially screened the compounds for their ability to influence the expression of occludin and ZO-1. An immunofluorescence screening was conducted to assess the ability of each compound to restore the expression of occludin and ZO-1, and the results were visualized as a heatmap (Figure 4C). The results showed that compound **10** significantly reversed the LPS-induced downregulation of these tight junction proteins in Caco-2 cells, and it was selected for further assessment of its anti-UC potential. Western blot analysis demonstrated that **10** dose-dependently enhanced the expression of tight junction proteins (E-cadherin and occludin) (Figure 4D). Supporting these findings, immunofluorescence analysis showed that **10** effectively reversed the LPS-induced downregulation of occludin and ZO-1 in Caco-2 cells (Figure 4E,F). Conventional UC medications such as 5-ASA and corticosteroids primarily exert therapeutic effects by suppressing excessive inflammation, and they are associated with significant adverse reactions, including gastrointestinal intolerance and systemic immunosuppression. More than one patient in ten ultimately requires surgical intervention [16,17]. However, compound **10** directly targets the structural restoration of the intestinal epithelial barrier without inducing obvious cytotoxicity, representing a promising lead compound for UC treatment.

## 3. Materials and Methods

### 3.1. General Experimental Procedures

Using a PerkinElmer 341 polarimeter (Hertford, UK), optical rotations were obtained. ECD spectra acquisition employed a Chirascan circular dichroism spectrometer (Applied Photophysics, Surrey, UK), while UV measurements utilized a Shimadzu UV-2600 UV−vis spectrophotometer (Shimadzu Corporation, Kyoto, Japan). For NMR analysis, both 1D and 2D spectra were acquired on Bruker AC 500 and 700 NMR spectrometers (Bruker, Fallanden, Switzerland), with TMS serving as the internal standard. X-ray diffraction intensity data were collected on an Agilent Xcalibur Nova single-crystal diffractometer using Cu Kα radiation (Agilent Technologies, Santa Clara, CA, USA). HRESIMS data collection occurred via a Bruker micro TOF-QII mass spectrometer (Fallanden, Switzerland), operating in either positive or negative ion mode. Thin layer chromatography (TLC) involved silica gel GF-254 (10–40 mm; Qingdao Marine Chemical Factory, Qingdao, China). Column chromatography (CC) relied upon a Sephadex LH-20 (Amersham Biosciences, Uppsala, Sweden) alongside silica gel (200–300 mesh and 100–200 mesh; Qingdao Marine Chemical Factory). HPLC separation was conducted on a Hitachi Primaide system equipped with a YMC ODS Series column (YMC-Pack ODS-A, 250 × 10 mm i.d., S-5 μm, 12 nm; YMC Co., Ltd., Kyoto, Japan). All solvents were of analytical grade (Tianjin Fuyu Chemical and Industry Factory, Tianjin, China), and both the fermentation culture medium and additional reagents originated from the Guangzhou Haili Aquarium Technology Company (Guangzhou, China).

### 3.2. Fungal Strain

From the South China Sea-collected green alga *Botryocladia* sp., the fungal strain *Penicillium stecki* SCSIO 41040 was isolated. This strain was subsequently preserved at 4 °C on Mueller–Hinton broth (MB) agar slants—containing malt extract (16 g), agar (18 g), sea-salt (30 g), and water (1 L) with pH 7.4–7.8—and ultimately archived at the CAS Key Laboratory of Tropical Marine Bio-Resources and Ecology, South China Sea Institute of Oceanology, Chinese Academy of Sciences (Guangzhou, China). Following PCR amplification of its ITS1-5.8S-ITS2 rDNA region (550 bp; GenBank: OP349656), DNA sequencing revealed 100% homology to *Penicillium steckii* (GenBank: NR111488).

### 3.3. Fermentation and Extraction

Into a 500 mL Erlenmeyer flask containing 150 mL seed medium (malt extract 1%, yeast extract 0.4%, glucose 0.4%, pH 7.2), several loops of strain SCSIO 41040 cells were inoculated. Subsequently, the seed culture was incubated at 28 °C for 48 h on a rotary shaker (180 rpm). For large-scale fermentation, 1 L conical flasks—each holding solid medium (300 mL/flask) prepared from KH_2_PO_4_ (0.5 g/L), MgSO_4_·7H_2_O (0.3 g/L), sorbitol (20 g/L), tryptophan (0.5 g/L), maltose (20 g/L), yeast extract (3 g/L), monosodium glutamate (10 g/L), and sea salt (32 g/L)—were inoculated and maintained at 25 °C. Following 32 days of cultivation, each flask’s fermented material underwent sequential EtOAc extraction (700 mL/flask), whereupon the combined extracts were suspended in MeOH. Petroleum ether was then employed to extract rice oil contaminants; ultimately, after concentration under reduced pressure, the MeOH solution yielded 40.0 g of reddish brown extract.

### 3.4. Isolation and Purification

The extract was subjected to silica gel column chromatography (CC), eluting with a gradient of CH_2_Cl_2_-MeOH (100:0–0:100) to give 15 fractions based on TLC properties. Initial purification of Fr.4, Fr.5, Fr.7, Fr.10, and Fr.11 was performed using ODS silica gel chromatography with MeOH/H_2_O (5–100%) as the eluent. Compound **14** (4.5 mg, *t*_R_ 12.1 min) was isolated from Fr.2 by semi-preparative HPLC (70% MeCN−H_2_O + 0.3% TFA, 2.5 mL/min). Compound **15** (4.5 mg, *t*_R_ 33.1 min) was isolated using HPLC from Fr.4.5 (25% MeCN−H_2_O + 0.3% TFA, 2.5 mL/min). Fr.7.3 was subjected to semi-preparative reversed-phase HPLC (62% MeOH−H_2_O + 0.3% TFA, 2.5 mL/min) to give four fractions. Fr.7.3.2 was processed through HPLC (60% MeOH/H_2_O + 0.3% TFA, 2.5 mL/min) to afford compounds **17** (48.0 mg, *t*_R_ 18.0 min), **20** (11.3 mg, *t*_R_ 22.0 min), and **26** (3.1 mg, *t*_R_ 25.2 min). The separation of Fr.7.4 via HPLC (32% MeCN−H_2_O + 0.3% TFA, 2.5 mL/min) delivered compound **1** (11.5 mg, *t*_R_ 15.2 min). Fr.10.5 was further purified by semi-preparative HPLC (45% MeCN−H_2_O + 0.3% TFA, 2.5 mL/min) to give five sub-fractions. Fr.10.5.5 was separated by semi-preparative HPLC (42% MeCN−H_2_O + 0.3% TFA, 2.5 mL/min) to give compounds **3** (3.3 mg, *t*_R_ 15.4 min) and **19** (3.7 mg, *t*_R_ 22.1 min). Then, **18** (4.0 mg, *t*_R_ 19.0 min) was afforded from Fr. 10.6 by HPLC (33% MeCN−H_2_O + 0.3% TFA, 2.5 mL/min). Fr.11.2 was further divided into six parts (Fr.11.2.1–Fr.11.2.6) through combiflash nextgen 300+ with MeOH. Fr.11.2.5 was purified by semi-preparative HPLC (30% MeCN/H_2_O + 0.3% TFA, 2.5 mL/min) to yield **12** (3.9 mg, *t*_R_ 15.0 min) and **24** (2.3 mg, *t*_R_ 8.8 min). Fr.11.2.6 was separated by semi-preparative HPLC (32% MeCN/H_2_O + 0.3% TFA, 2.5 mL/min) to provide **4** (1.8 mg, *t*_R_ 16.0 min) and **10** (14.0 mg, *t*_R_ 8.0 min). Fr.11.3 was subjected to semi-preparative reversed-phase HPLC (60% MeOH−H_2_O + 0.3% TFA, 2.0 mL/min) to give **25** (3.2 mg, *t*_R_ 25.0 min) and an additional four fractions (Fr.11.3.1–Fr.11.3.4). Fr.11.3.2 was further purified by semi-preparative HPLC (36% MeCN−H_2_O, 2.5 mL/min) to give compound **23** (5.4 mg, *t*_R_ 15.3 min). The purification of Fr.11.3.3 by HPLC (36% MeCN/H_2_O, 2.5 mL/min) yielded compounds **11** (2.7 mg, *t*_R_ 19.8 min), **5** (1.3 mg, *t*_R_ 17.8 min), and **13** (3.3 mg, *t*_R_ 27.5 min). Compound **21** (6.9 mg, *t*_R_ 16.0 min) and five sub-fractions (Fr.11.4.1–Fr.11.4.5) of component Fr.11.4 were obtained by semi-preparative reversed-phase high-performance liquid chromatography (68% MeCN−H_2_O + 0.3% TFA, 2.5 mL/min). Compound **16** (2.8 mg, *t*_R_ 14.0 min) was isolated using HPLC from Fr.11.4.2 (47% MeCN/H_2_O, 2.5 mL/min). From Fr.11.4.3, compound **6** (6.3 mg, *t*_R_ 34.0 min) was acquired following HPLC (68% MeOH/H_2_O, 2.5 mL/min). Compounds **2** (1.8 mg, *t*_R_ 18.3 min) and **22** (9.3 mg, *t*_R_ 13.0 min) were isolated from Fr.11.4.5 by HPLC (62% MeOH−H_2_O + 0.3% TFA, 2.5 mL/min). Fr.11.5 was fractionated by HPLC (70% MeOH−H_2_O + 0.3% TFA, 2.5 mL/min), yielding compound **7** (20.0 mg, *t*_R_ 27.0 min) and four sub-fractions (Fr.11.5.1–Fr.11.5.4). Subsequently, compounds **8** (14.0 mg, *t*_R_ 29.0 min) and **9** (52.0 mg, *t*_R_ 21.4 min) were isolated from Fr.11.5.3 and Fr.11.5.4 via HPLC (67% MeOH/H_2_O, 2.5 mL/min; 70% MeOH/H_2_O, 2.5 mL/min), respectively.

**1**: Yellow powder; ${\left[\alpha \right]}_{\;D}^{25}$ + 30.9 (*c*, 0.1, MeOH); UV (MeOH) *λ*_max_ (log *ε*) 263 (4.17); ECD (0.130 mM, MeOH) *λ*_max_ (Δ*ε*) 268 (+7.90) nm; ^1^H and ^13^C NMR data, Table 1; HRESIMS *m*/*z* 305.1762 [M − H]^−^ (calcd for C_18_H_25_O_4_^−^, 305.1758).

**2**: Yellow powder; ${\left[\alpha \right]}_{\;D}^{25}$ + 22.1 (*c*, 0.1, MeOH); UV (MeOH) *λ*_max_ (log *ε*) 262 (4.27); ECD (0.131 mM, MeOH) *λ*_max_ (Δ*ε*) 257 (-23.79), 288 (+23.29) nm; ^1^H and ^13^C NMR data, Table 1; HRESIMS *m*/*z* 305.1763 [M − H]^−^ (calcd for C_18_H_25_O_4_^−^, 305.1758).

**3**: Yellow oil; ${\left[\alpha \right]}_{\;D}^{25}$ + 35.4 (*c*, 0.1, MeOH); UV (MeOH) *λ*_max_ (log *ε*) 200 (3.91); ECD (0.130 mM, MeOH) *λ*_max_ (Δ*ε*) 200 (−30.00) nm; ^1^H and ^13^C NMR data, Table 1; HRESIMS *m*/*z* 307.1912 [M − H]^−^ (calcd for C_18_H_27_O_4_^−^, 307.1915).

Crystal data for C_36_H_48_O_8_ (M = 608.74 g/mol) (**8**): monoclinic, space group P2_1_ (no. 4), *a* = 7.03460(10) Å, *b* = 12.6237(2) Å, *c* = 18.8721(3) Å, *β* = 89.1090(10)°, *V* = 1675.69(4) Å^3^, *Z* = 2, *T* = 100.00(10) K, μ(Cu Kα) = 0.680 mm^−1^, *Dcalc* = 1.206 g/cm^3^, 31,927 reflections measured (8.428° ≤ 2Θ ≤ 148.944°), 6691 unique (*R*_int_ = 0.0597, *R*_sigma_ = 0.0320), which were used in all calculations. The final *R*_1_ was 0.0377 (I > 2σ(I)) and *wR*_2_ was 0.1049 (all data).

Crystal data for C_16_H_22_O_4_ (M = 278.33 g/mol) (**22**): orthorhombic, space group P2_1_2_1_2_1_ (no. 19), *a* = 5.12820(10) Å, *b* = 11.6636(2) Å, *c* = 24.1513(3) Å, *V* = 1444.57(4) Å^3^, *Z* = 4, *T* = 99.98(14) K, μ(Cu Kα) = 0.739 mm^−1^, *Dcalc* = 1.280 g/cm^3^, 13,864 reflections measured (7.32° ≤ 2Θ ≤ 148.872°), 2896 unique (*R*_int_ = 0.0426, *R*_sigma_ = 0.0301), which were used in all calculations. The final *R*_1_ was 0.0314 (I > 2σ(I)) and *wR*_2_ was 0.0795 (all data).

Crystal data for C_16_H_24_O_3_ (M = 264.35 g/mol) (**23**): monoclinic, space group I_2_ (no. 5), *a* = 12.72599(11) Å, *b* = 5.50132(5) Å, *c* = 22.58030(18) Å, *β* = 101.3861(8)°, *V* = 1549.73(2) Å^3^, *Z* = 4, *T* = 99.98(12) K, μ(Cu Kα) = 0.611 mm^−1^, *Dcalc* = 1.133 g/cm^3^, 15,187 reflections measured (7.416° ≤ 2Θ ≤ 148.936°), 3088 unique (*R*_int_ = 0.0257, *R*_sigma_ = 0.0186), which were used in all calculations. The final *R*_1_ was 0.0263 (I > 2σ(I)) and *wR*_2_ was 0.0667 (all data).

Compounds **8**, **22**, and **23** were crystallized from slow evaporation in a CH_3_OH solution (Figure S33). A single crystal with the indicated dimensions was selected and measured on an Agilent Xcalibur Nova single-crystal diffractometer using Cu K*α* radiation and refined by full-matrix least-squares calculation. The crystallographic data for structures **8**, **22**, and **23** have been deposited with the Cambridge Crystallographic Data Centre with supplementary publication numbers CCDC-2499249, CCDC-2499251, and CCDC-2499252, respectively.

### 3.5. ECD Calculation

The new compounds underwent random conformational searches using Spartan’14 (with MMFF) and Gaussian 09 (with DFT/TDDFT). The MMFF search first yielded low-energy conformers (Boltzmann population > 5%), which were then geometrically optimized via DFT (B3LYP/6-311G*) in MeOH using the IEFPCM model. For these stable conformers, ECD calculations were further performed in MeOH using TDDFT at the B3LYP/6-311G* level. Finally, Multiwfn (0.2–0.4 eV half-bandwidth) generated the ECD spectra, and the Boltzmann-weighted contributions were calculated after UV simulation.

### 3.6. Cytotoxic Activity Measurement

Caco-2 cells were seeded at a density of 4 × 10^3^ cells/well in 96-well plates. After treating the Caco-2 cells with different concentrations of tanzawaic acids for 24 h, 10 μL CCK-8 (PWL111, Bgbioscience, Dalian, China) test reagent was added to each well, following the manufacturer’s instructions [25,28].

### 3.7. Caco-2 and THP-1 Co-Culture Model Incubation

In brief, Caco-2 cells were seeded onto Transwell insert plates at a concentration of 1.5 × 10^5^ cells/cm^2^ and cultured for 21 days until cells were fully differentiated. The culture medium was changed every 2–3 days. THP-1 were seeded onto 6-well plates (4 × 10^6^ cells/well) and rested for 24 h. After replacing the media with complete DMEM, inserts with Caco-2 were added into plates containing THP-1. One microgram per milliliter of LPS was added to the basolateral side, and, after 3 h of incubation, different concentrations of tanzawaic acids (10 μM) were added to the apical side of the insert. After 24 h of incubation, Caco-2 cells from the insert were collected.

### 3.8. Western Blot Analysis

Caco-2 cells were lysed with ice-cold radioimmunoprecipitation assay (RIPA) lysis buffer (P0013B; Beyotime Biotechnology, Shanghai, China) supplemented with protease and phosphatase inhibitors, and the lysates were centrifuged at 14,000 r/min for 15 min. The protein concentrations were measured with a bicinchoninic acid (BCA) kit (23,227; Thermo Fisher Scientific, Waltham, MA, USA), and the protein samples were prepared using 4× sodium dodecyl sulfate–polyacrylamide gel electrophoresis (SDS-PAGE) loading buffer. Equal amounts of proteins were loaded onto 10% SDS-PAGE gels, and the separated proteins were transferred to polyvinylidene difluoride (PVDF) membranes (3010040001; Roche Diagnostics, Basel, Switzerland). The membranes were blocked with 5% skim milk in tris-buffered saline with Tween 20 (TBST) and incubated with the indicated primary antibodies at 4 °C overnight. The membranes were then washed and incubated with a horseradish peroxidase (HRP)-conjugated secondary antibody for 1 h at room temperature. Finally, the bands were detected using an enhanced chemiluminescence (ECL) kit (P0018HM; Beyotime Biotechnology, China). The antibodies used for Western blot analysis were occludin (Servicebio, GB111401-100, 1:1000, Wuhan, China), E-cadherin (Abcam, ab76055, 1:1000, Cambridge, UK), and Gapdh (Abmart, P60037; 1:3000, Shanghai, China).

### 3.9. Immunofluorescence

In brief, Caco-2 cells were fixed with 4% paraformaldehyde for 15 min at room temperature and washed three times with cold PBS for 3 min each. Treatment with 0.5% Triton-X 100 was performed for 20 min at room temperature, followed by three additional washes with cold PBS for 3 min each. The cells were then blocked with 10% goat serum for 1 h at room temperature. Subsequently, the blocking solution was removed, and the cells were incubated overnight at 4 °C with the occludin (Servicebio, GB111401-100, 1:200) and ZO-1 (Servicebio, GB111402-100, 1:200) primary antibody. On the following day, the antibody was removed, and the cells were washed three times with PBS for 3 min each. FITC-AffiniPuro Goat Anti-Mouse IgG (Yeasen, 33207ES60, 1:100, Shanghai, China) was added, and cells were incubated in the dark at 37 °C for 1 h, followed by three washes with PBS for 3 min each. Finally, 5 μg/mL DAPI was added, and cells were incubated at room temperature for 5 min and washed three times with PBS for 5 min each. Fluorescence imaging was conducted using a laser confocal microscope (TCSSP8 DIVE, Leica, Wetzlar, Germany).

## 4. Conclusions

Marine-derived fungi have emerged as an underexplored reservoir of structurally unique secondary metabolites with diverse pharmacological activity. Tanzawaic acids, a class of decalin-containing polyketides, have previously been reported to exhibit antibacterial, antifungal, and anti-inflammatory properties. However, to the best of our knowledge, this is the first systematic investigation of their intestinal barrier-protective activity, which significantly expands the therapeutic potential of this compound class. In this study, we demonstrated that tanzawaic acids, particularly compound **10**, could effectively reverse the LPS-induced downregulation of ZO-1 and occludin, thereby restoring intestinal barrier integrity. However, several limitations of this study should be acknowledged. First, only in vitro activity was evaluated, and the in vivo therapeutic efficacy of compound **10** remains to be verified in animal models such as dextran sulfate sodium (DSS)-induced colitis. Second, the detailed molecular mechanisms underlying its barrier-protective effect, including specific signaling pathways, have not been fully elucidated. These unresolved questions will be the focus of our ongoing and future research efforts.

In summary, a further study of a marine-derived *Penicillium steckii* revealed three new (**1**–**3**) and twenty-three known (**4**–**26**) tanzawaic acids, which were obtained from the strain fermented on a liquid medium. Extensive NMR spectroscopic and HRESIMS data and ECD and NMR calculations were used to determine the structures of the new compounds. Based on the LPS-induced Caco-2/THP-1 cell injury model, the protective role of tanzawaic acids in inflammatory damage to the intestinal epithelial barrier was measured. Notably, compound **10** enhanced the expression and membrane localization of ZO-1 and occludin, which contributed to the restoration of barrier integrity. This study not only enriches the structural diversity of tanzawaic acids but also identifies compound **10** as a potential lead compound for UC treatment. Further in vivo efficacy studies, mechanistic investigations, and structural optimizations are warranted to translate these findings into clinical applications.

## Figures and Tables

**Table 1:** ^1^H and ^13^C NMR spectroscopic data of **1**–**3** (500, 125 MHz, DMSO-*d*_6_).

No.	1		2		3	
	δ_C_, Type	δ_H_ (J in Hz)	δ_C_, Type	δ_H_ (J in Hz)	δ_C_, Type	δ_H_ (J in Hz)
1	168.5, C		168.5, C		173.6, C	
2	122.0, CH	5.76, d (15.2)	122.4, CH	5.82, d (15.4)	67.9, CH	4.24, m
3	143.4, CH	7.10, dd (15.2, 9.1)	142.8, CH	7.12, dd (15.4, 9.8)	43.3, CH_2_	2.19, m
						2.20, m
4	126.8, CH	6.17, m	128.1, CH	6.15, m	130.8, CH	5.32, m
5	148.3, CH	6.17, d (15.0)	147.2, CH	6.11, d (15.4)	134.4, CH	5.61, dd (15.7, 9.8)
6	48.6, CH	2.37, m	55.8, CH	1.99, m	47.9, CH	2.22, m
7	44.6, CH	0.95, m	49.2, CH	1.40, m	45.2, CH	0.78, m
8	30.9, CH	1.62, m	36.7, CH	1.30, m	31.7, CH	1.62, m
9	48.8, CH_2_	1.09, m	49.2, CH_2_	0.76, m	50.1, CH_2_	1.02, m
		1.54, m		1.57, m		1.49, m
10	70.4, C		30.6, CH	1.34, m	67.9, C	
11	76.4, CH	2.81, d (10.9)	38.7, CH_2_	0.74, m	45.8, CH_2_	1.04, m
				2.14, m		1.54, m
12	43.3, CH	2.07, m	50.6, CH	1.25, m	37.3, CH	2.19, m
13	129.3, CH	5.93, d (9.6)	77.3, CH	3.88, d (11.4)	131.9, CH	5.32, m
14	132.2, CH	5.59, dd (9.6, 3.4)	210.4, C		132.5, CH	5.53, dt (9.5, 3.6)
15	36.1, CH	2.09, m	44.8, CH	2.44, m	37.1, CH	2.02, m
16	16.3, CH_3_	0.90, d (6.4)	12.4, CH_3_	0.82, d (6.6)	16.3, CH_3_	0.86, d (7.1)
17	27.4, CH_3_	1.08, s	22.3, CH_3_	0.87, d (6.4)	31.3, CH_3_	1.07, s
18	21.8, CH_3_	0.81, d (7.0)	23.9, CH_3_	0.94, d (6.3)	22.6, CH_3_	0.88, d (6.4)

## Data Availability

The original contributions presented in this study are included in the article.

## Supplementary Materials

The following supporting information can be downloaded at https://www.mdpi.com/article/10.3390/md24050147/s1. Figures S1–S32: ^1^H, ^13^C, DEPT135, COSY, HSQC, HMBC, NOESY, HRESIMS, UV spectra, CD spectra, and DP4^+^ analysis of the new compounds **1**–**3**; Figure S33 X-ray crystal structures of 8, 22 and 23; Figure S34 The original untreated photograph of the Western blot gel.
