# Supplementary material for: Anti-Ulcerative Colitis Tanzawaic Acids from a Marine Algicolous Fungus, Penicillium steckii SCSIO 41040
Source: Mar Drugs. 2026 Apr 22;24(5):147. doi: 10.3390/md24050147 (PMC13208603; doi:10.3390/md24050147)
Supplement: Supplementary file 1 [file marinedrugs-24-00147-s001.zip › marinedrugs-4255900-supplementary.pdf]

Supporting Information

Anti-Ulcerative Colitis Tanzawaic Acids from a Marine Algicolous Fungus *Penicillium steckii* SCSIO41040

Yingying Song [1 †], Jiamin Wang [2 †], Yuchen Lin [2], Jianglian She [3, 4], Yalin Liu [1], Xiangxi Yi [1], Chenghai Gao [1 *], Junfeng Wang [3, 4 *], and Yonghong Liu [1, 3, 4 *]

[1] Guangxi Key Laboratory of Marine Drugs, University Engineering Research Center of High-Efficient Utilization of Marine Traditional Chinese Medicine Resources, Guangxi, Institute of Marine Drugs/Faculty of Pharmacy, Guangxi University of Chinese Medicine, Nanning 530200, China; 16637610987@163.com (Y.S.); lyl20000627@163.com (Y.L.); yixiangxi2017@163.com (X.Y.).

[2] Department of Pathophysiology, School of Medicine, Jinan University, Guangzhou 510632, China; wjm2275322055@163.com (J.W.); linyuchen9713@163.com (Y.L.).

[3] Guangdong Key Laboratory of Marine Materia Medica, State Key Laboratory of Tropical Oceanography, South China Sea Institute of Oceanology, Chinese Academy of Sciences, Guangzhou 510301, China; shejianglian20@mails.ucas.ac.cn (J.S.).

[4] University of Chinese Academy of Sciences, 19 Yuquan Road, Beijing 100049, China

* Correspondence: gaoch@gxtcmu.edu.cn (C.G.); wangjunfeng@scsio.ac.cn (J.W.); yonghongliu@scsio.ac.cn (Y.L.)

† These authors contributed equally to this work.

List of Supporting Information

The ITS gene sequence data of *Penicillium steckii.* SCSIO 41040
Figure S1 ^{1}H NMR spectrum of compound **1** in DMSO-d_6
Figure S2 ^{13}C NMR spectrum of compound **1** in DMSO-d_6
Figure S3 DEPT135 spectrum of compound **1** in DMSO-d_6
Figure S4 ^{1}H-^{1}H COSY spectrum of compound **1** in DMSO-d_6
Figure S5 HSQC spectrum of compound **1** in DMSO-d_6
Figure S6 HMBC spectrum of compound **1** in DMSO-d_6
Figure S7 NOESY spectrum of compound **1** in DMSO-d_6
Figure S8 HRESIMS spectrum of compound **1**
Figure S9 UV spectrum of **1**
Figure S10 CD spectrum of **1**

Figure S11 ^{1}H NMR spectrum of compound **2** in DMSO-d_6
Figure S12 ^{13}C NMR spectrum of compound **2** in DMSO-d_6
Figure S13 DEPT135 spectrum of compound **2** in DMSO-d_6
Figure S15 ^{1}H-^{1}H COSY spectrum of compound **2** in DMSO-d_6
Figure S16 HSQC spectrum of compound **2** in DMSO-d_6
Figure S17 HMBC spectrum of compound **2** in DMSO-d_6
Figure S18 NOESY spectrum of compound **2** in DMSO-d_6
Figure S19 HRESIMS spectrum of compound **2**
Figure S20 UV spectrum of **2**
Figure S21 CD spectrum of **2**

Figure S22 ^{1}H NMR spectrum of compound **3** in DMSO-d_6
Figure S23 ^{13}C NMR spectrum of compound **3** in DMSO-d_6
Figure S24 DEPT135 spectrum of compound **3** in DMSO-d_6
Figure S25 ^{1}H-^{1}H COSY spectrum of compound in DMSO-d_6
Figure S26 HSQC spectrum of compound **3** in DMSO-d_6
Figure S27 HMBC spectrum of compound **3** in DMSO-d_6
Figure S28 NOESY spectrum of compound **3** in DMSO-d_6
Figure S29 HRESIMS spectrum of compound **3**
Figure S30 UV spectrum of **3**
Figure S31 CD spectrum of **3**
Figure S32 DP4^{+} analysis of calculated ^{13}C NMR data of **3**
Figure S33 X-ray crystal structures of **8**, **22** and **23**
Figure S34 The original untreated photograph of the Western blot gel

The ITS gene sequence data of *Penicillium steckii* sp. SCSIO 41040

GATATGCTTAAGTTCAGCGGGTATCCCTACCTGATCCGAGGTCAACCTGAGAAAAAATA
AAAGGTTGGGGGTCGGCTGGCGCCGGCCGGGCCTACAAGAGCGGGTGACGAAGCCCC
ATACGCTCGAGGACCGGACGCGGTGCCGCCGCTGCCTTTCGGGCCCGTCCCCCCCGGA
GCGGGGGGGACGGGGCCCAACACACAAGCCGTGCTTGAGGGCAGCAATGACGCTCG
GACAGGCATGCCCTCCGGAATACCAGAGGGCGCAATGTGCGTTCAAAGACTCGATGAT
TCACTGAATTCTGCAATTCACATTAGTTATCGCATTTCGCTGCGTTCTTCATCGATGCCG
GAACCAAGAGATCCGTTGTTGAAAGTTTTAACTAATTTAGCTAGTTGTCTCAGACTGCA
ACTTCAGACAGCGTTCAGAGGGGGGCTTCGGCGGGCGCGGGCCCGGGGGCGGATGCC
CCCCGGCGGCCTGGCGGCGGGCCCGCCGAAGCAACACAGGTTCGTGCAACACGGGTG
GGAGGTTGGACCCAGAGGGCCCTCACTCGGTAATGATCCTTCCGCAG

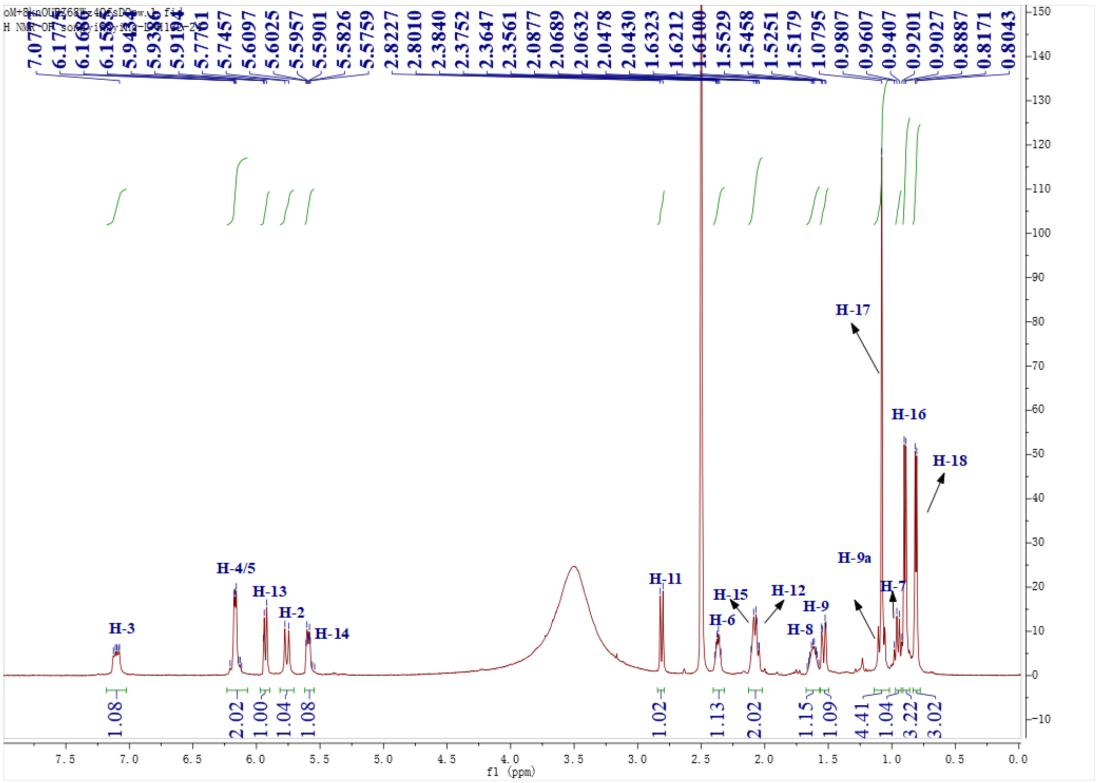

Figure S1 ^{1}H NMR spectrum of compound **1** in DMSO-d_6

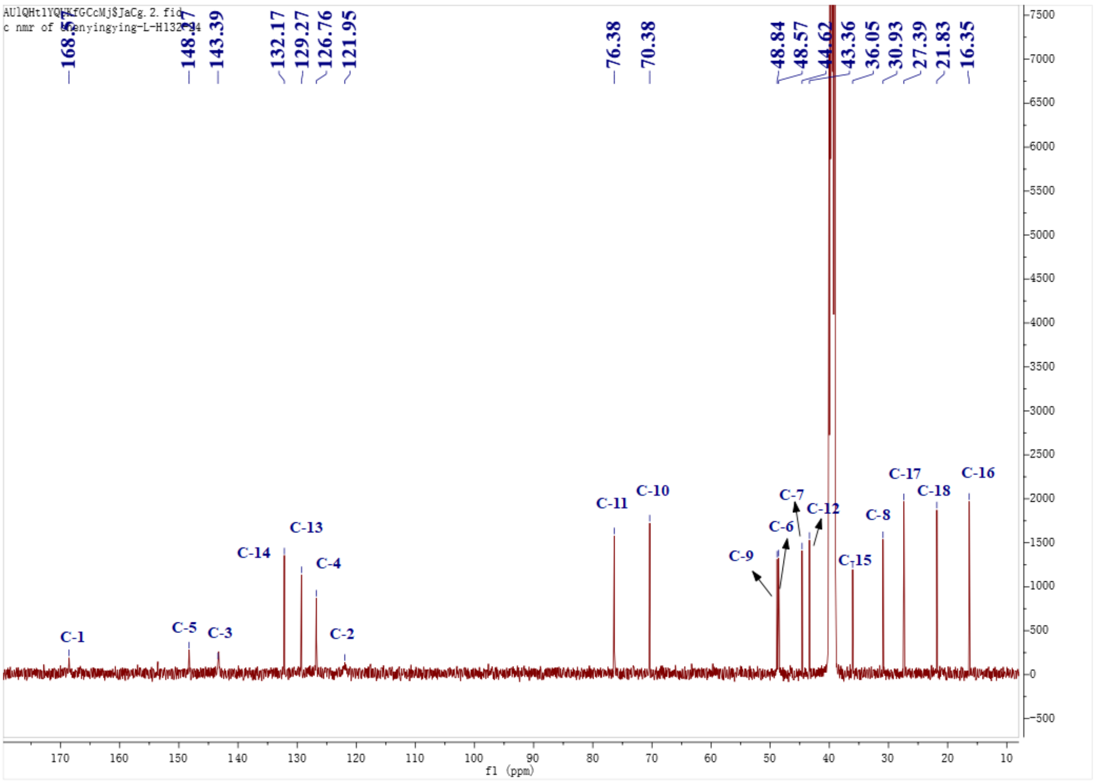

Figure S2 ^{13}C NMR spectrum of compound **1** in DMSO-d_6

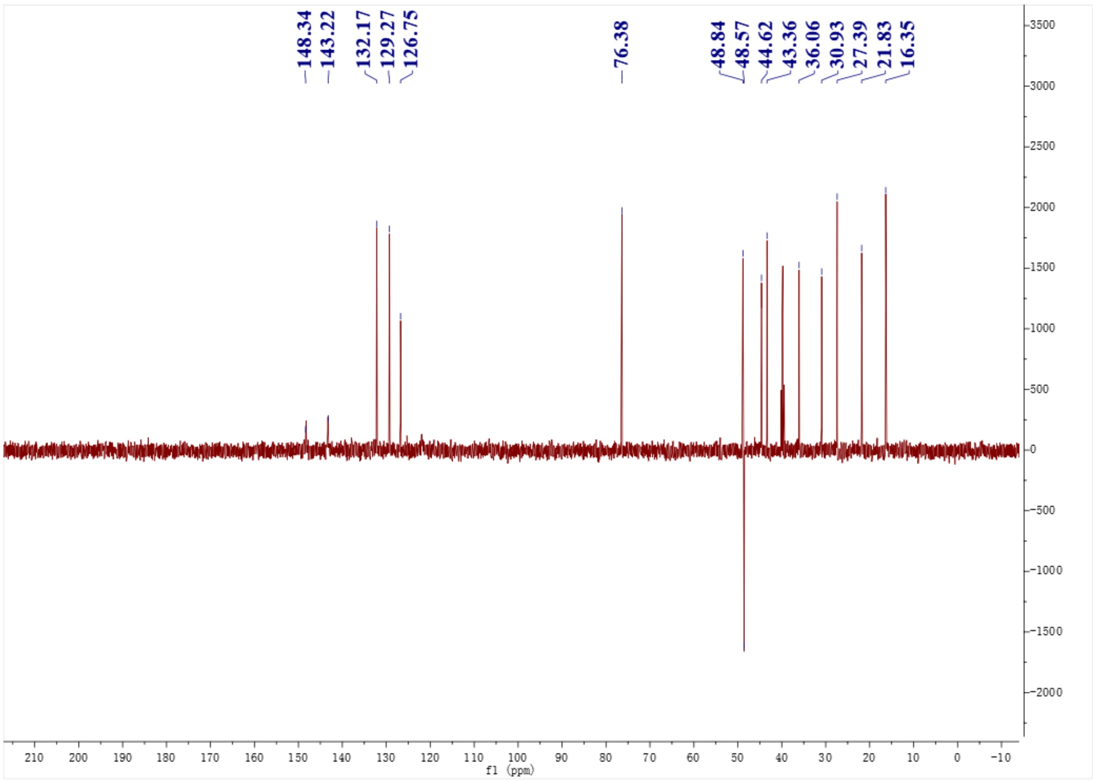

Figure S3 DEPT135 spectrum of compound **1** in DMSO-d_6

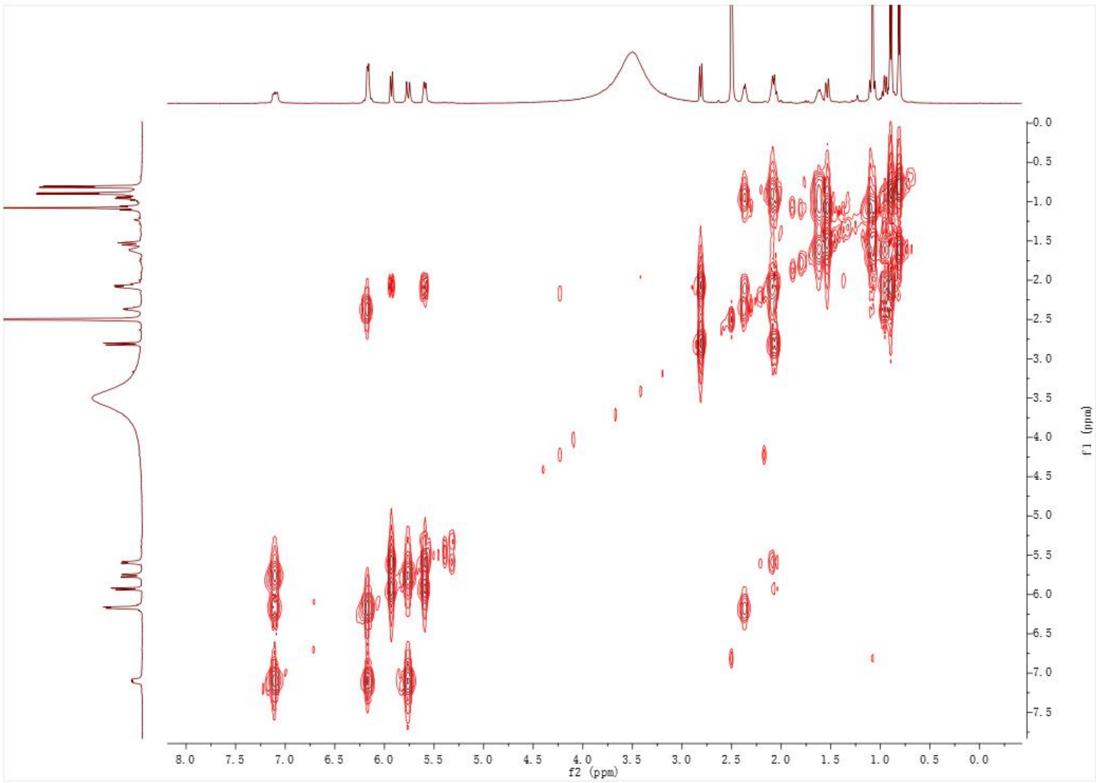

Figure S4 ^{1}H-^{1}H COSY spectrum of compound **1** in DMSO-*d*$_6$

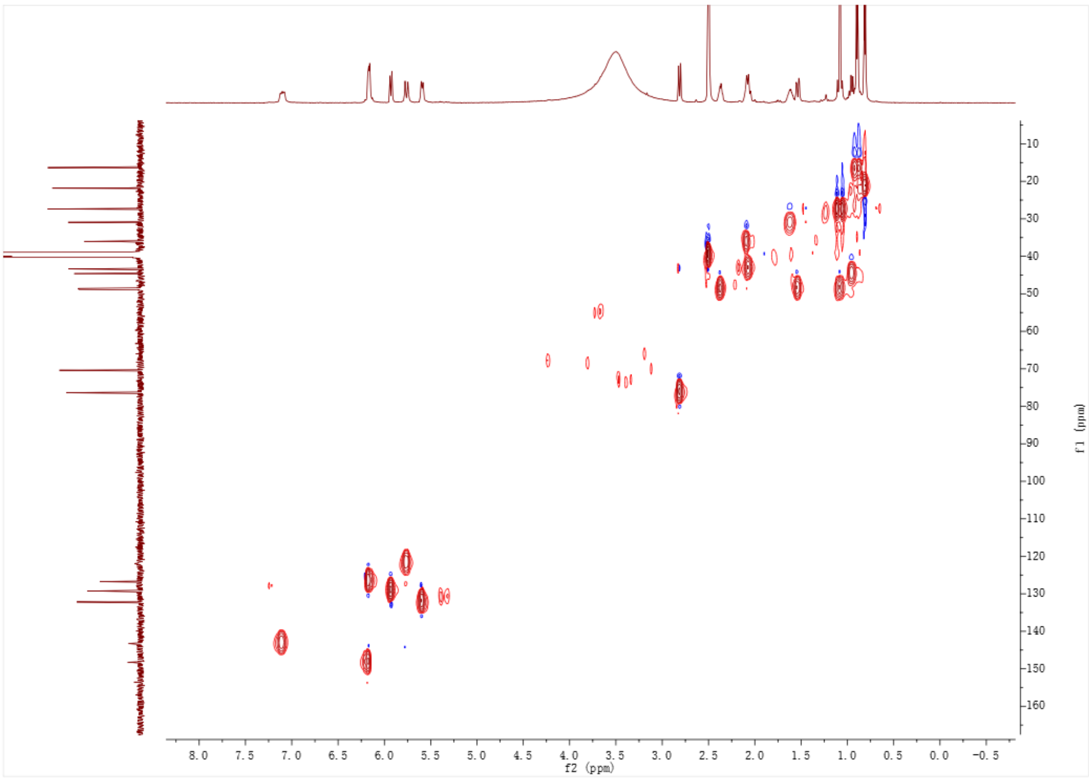

Figure S5 HSQC spectrum of compound **1** in DMSO-*d*$_6$

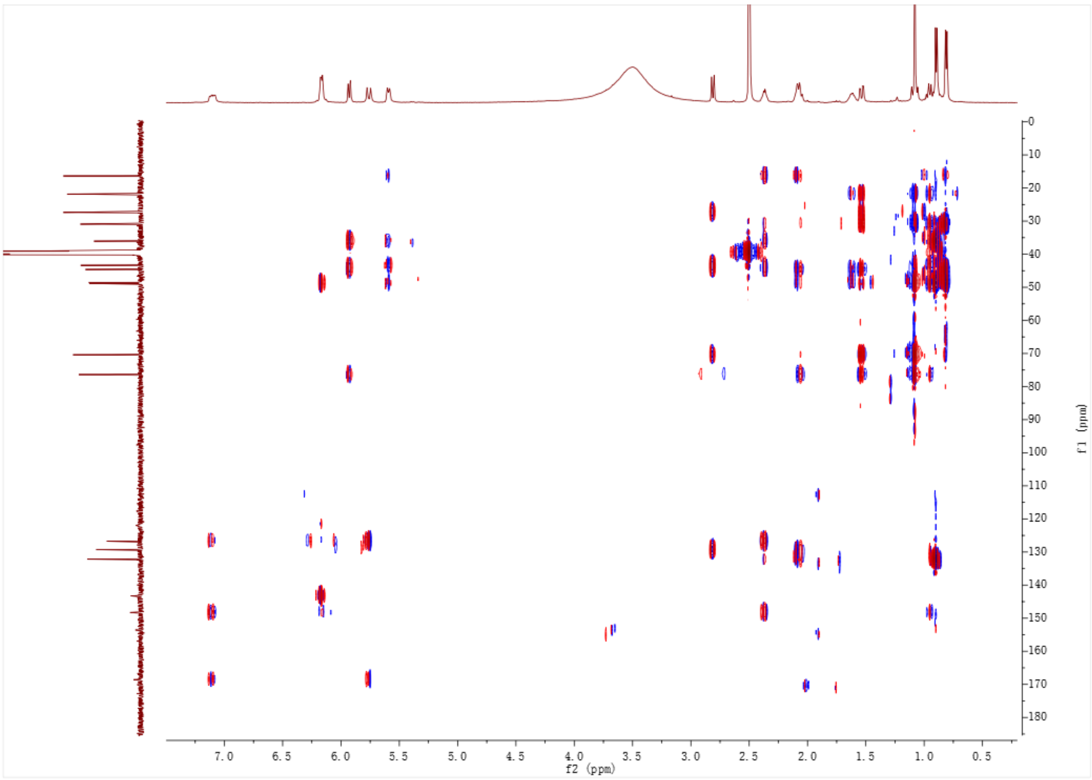

Figure S6 HMBC spectrum of compound **1** in DMSO-d_6

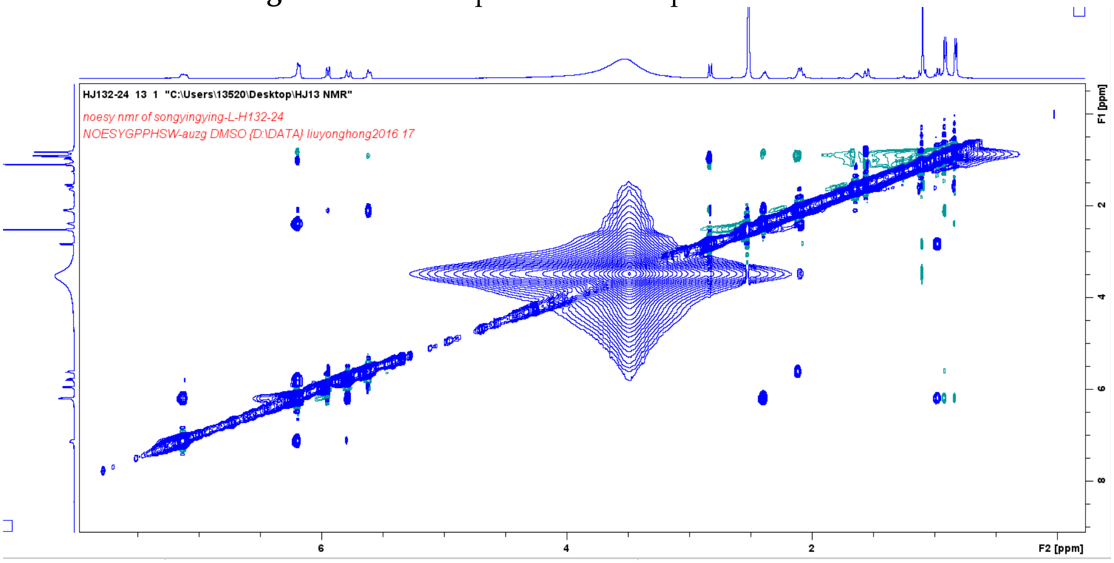

Figure S7 NOESY spectrum of compound **1** in DMSO-d_6

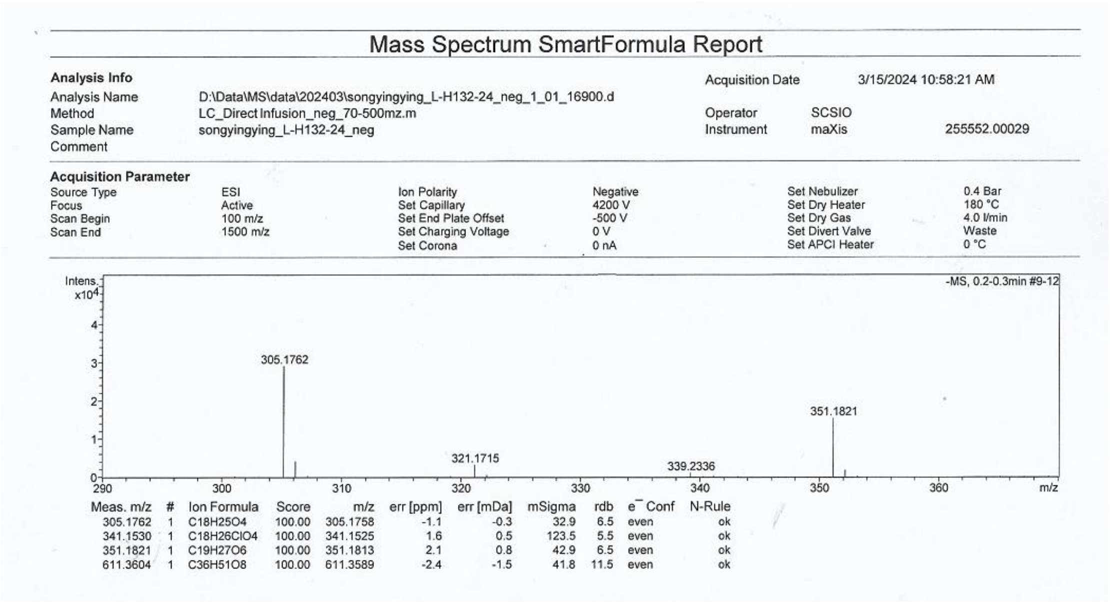

Meas. m/z	#	Ion Formula	Score	m/z	err [ppm]	err [mDa]	mSigma	rdb	e⁻ Conf	N-Rule
305.1762	1	C18H25O4	100.00	305.1758	-1.1	-0.3	32.9	6.5	even	ok
341.1530	1	C18H26ClO4	100.00	341.1525	1.6	0.5	123.5	5.5	even	ok
351.1821	1	C19H27O6	100.00	351.1813	2.1	0.8	42.9	6.5	even	ok
611.3604	1	C36H51O8	100.00	611.3589	-2.4	-1.5	41.8	11.5	even	ok

Figure S8 HRESIMS spectrum of compound **1**

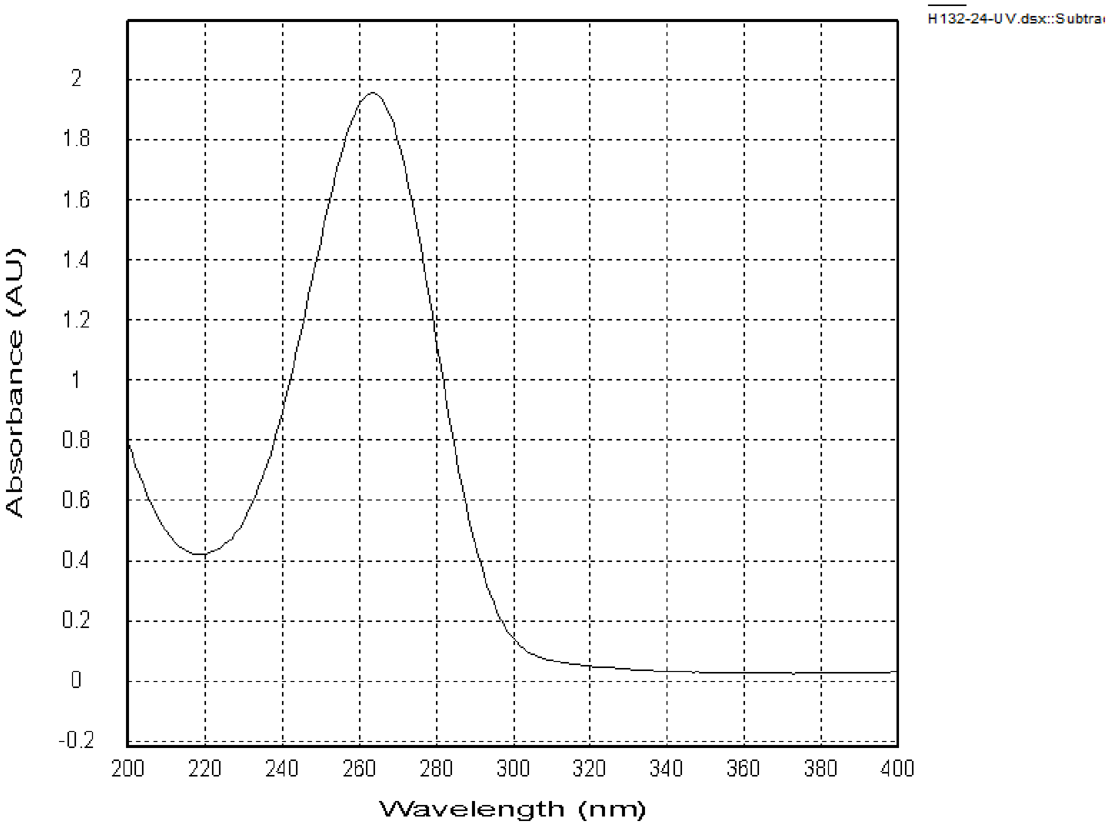

Figure S9 UV spectrum of **1**

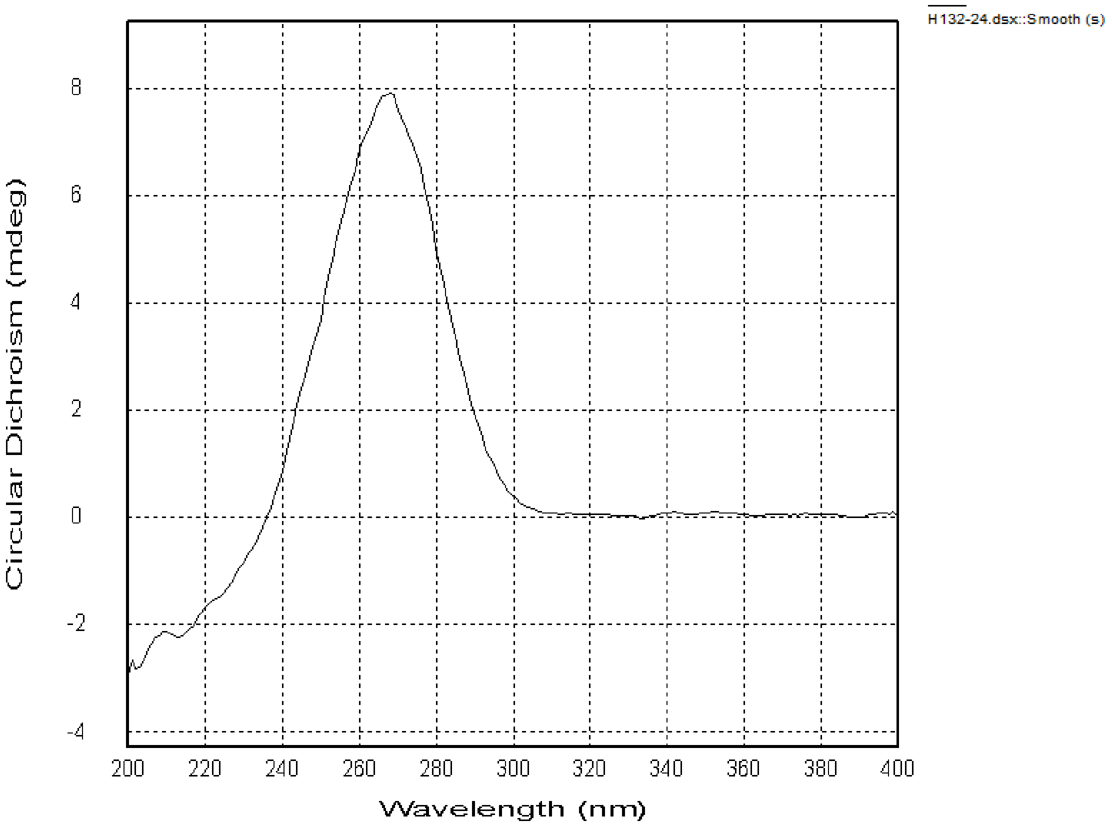

Figure S10 CD spectrum of **1**

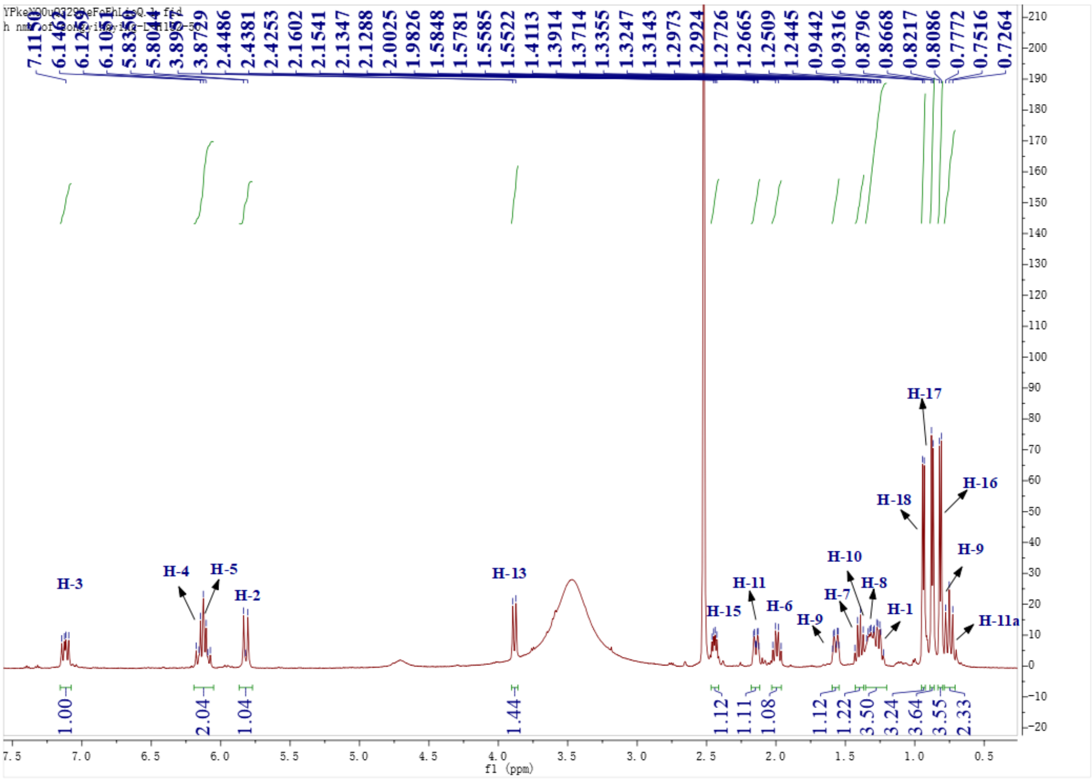

Figure S11 ^{1}H NMR spectrum of compound **2** in DMSO-d_6

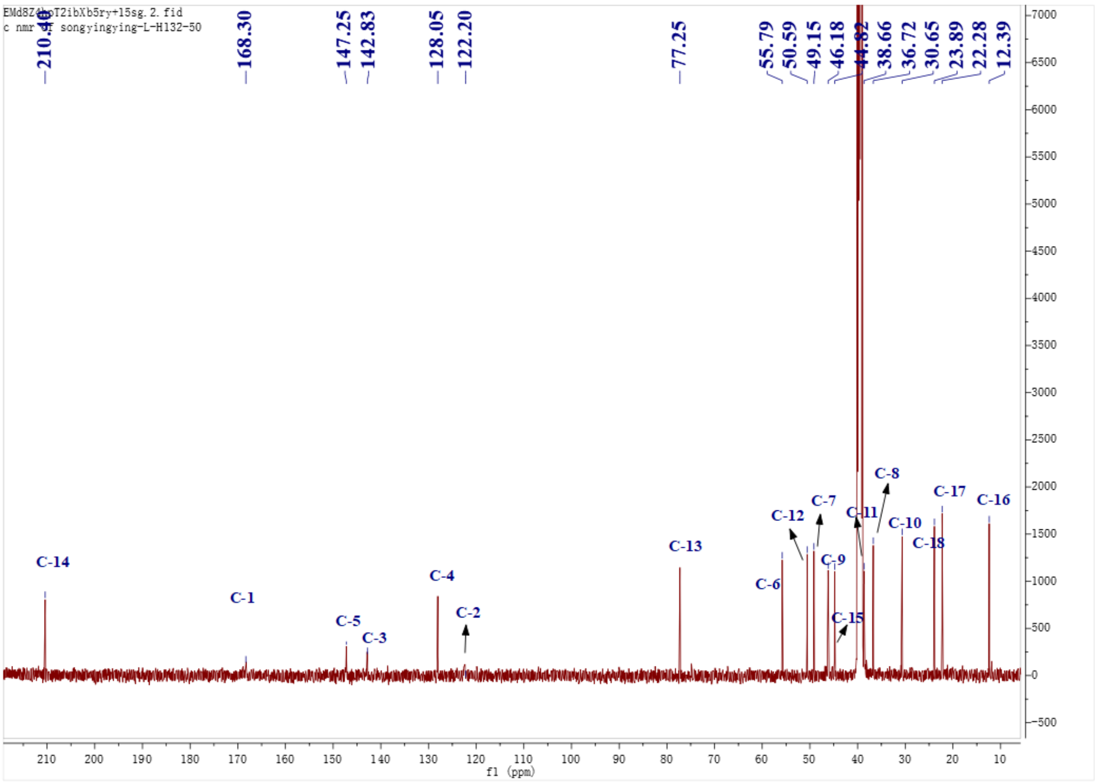

Figure S12 ^{13}C NMR spectrum of compound **2** in DMSO-d_6

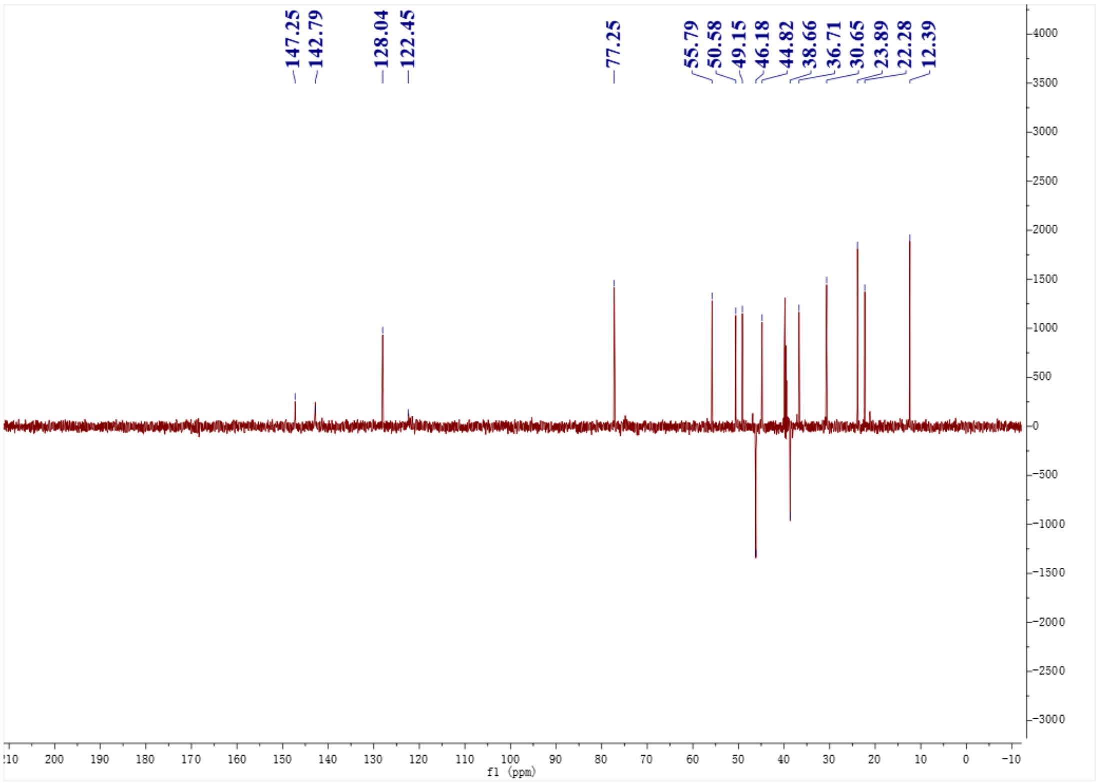

Figure S13 DEPT135 spectrum of compound **2** in DMSO-d_6

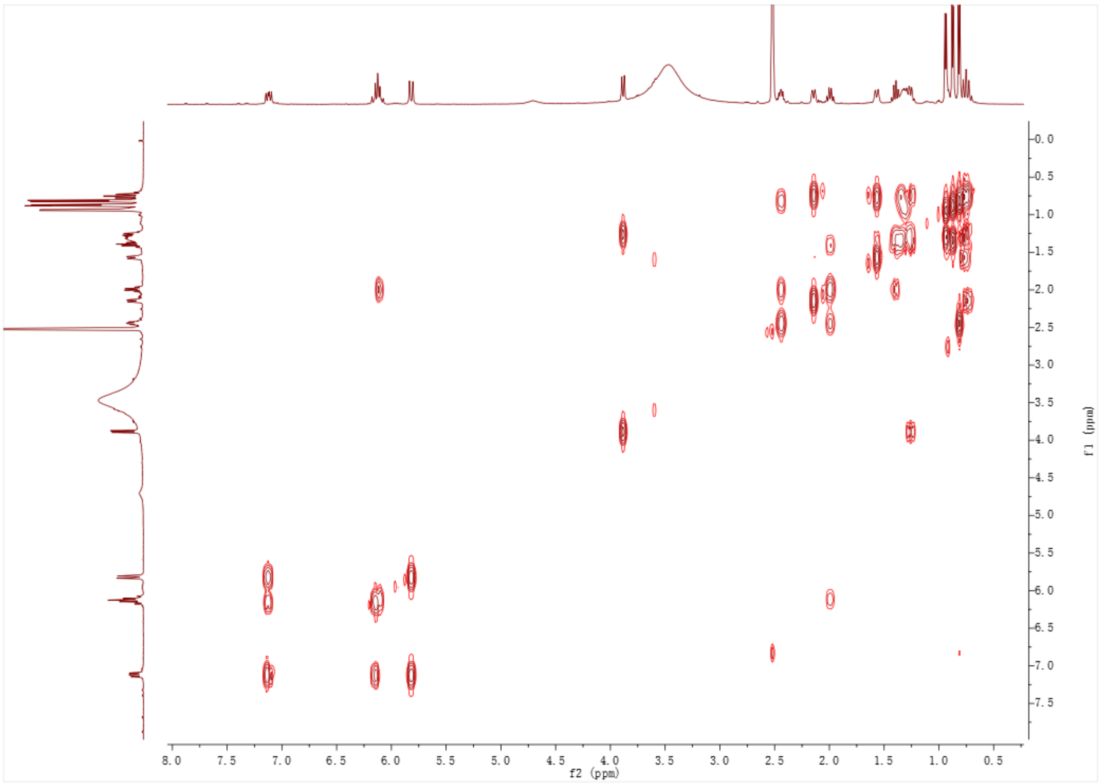

Figure S15 ^{1}H-^{1}H COSY spectrum of compound **2** in DMSO-*d*$_6$

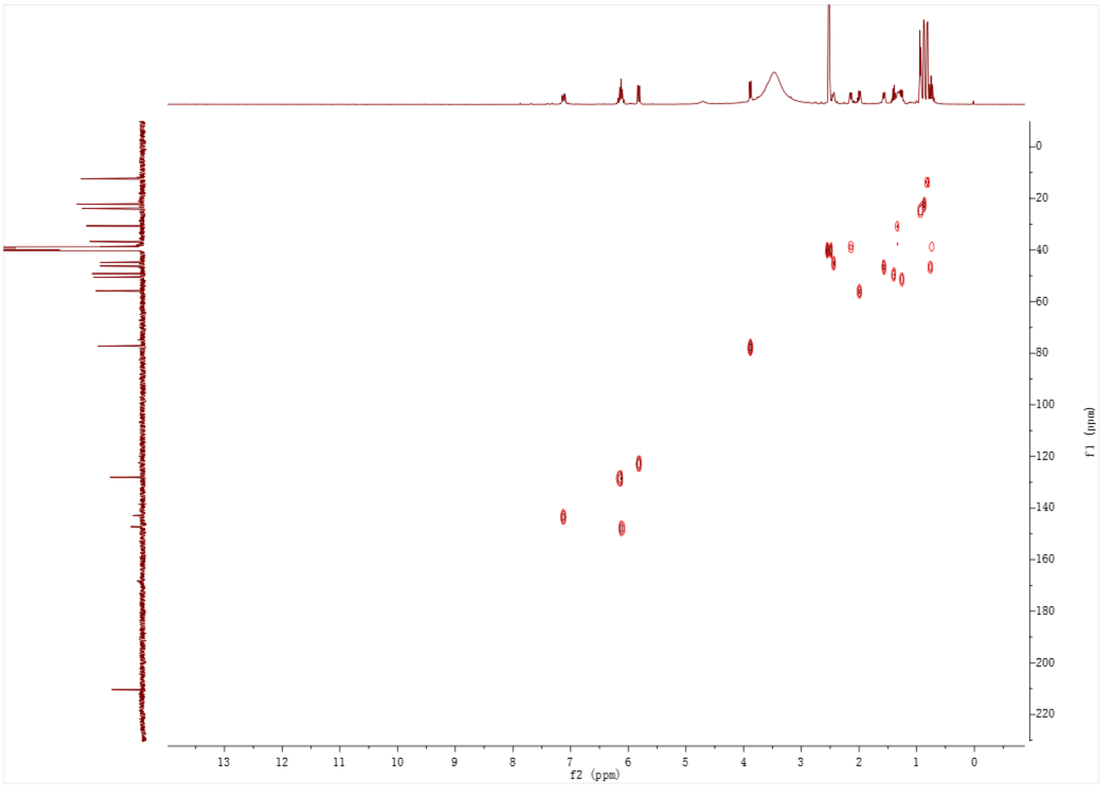

Figure S16 HSQC spectrum of compound **2** in DMSO-*d*$_6$

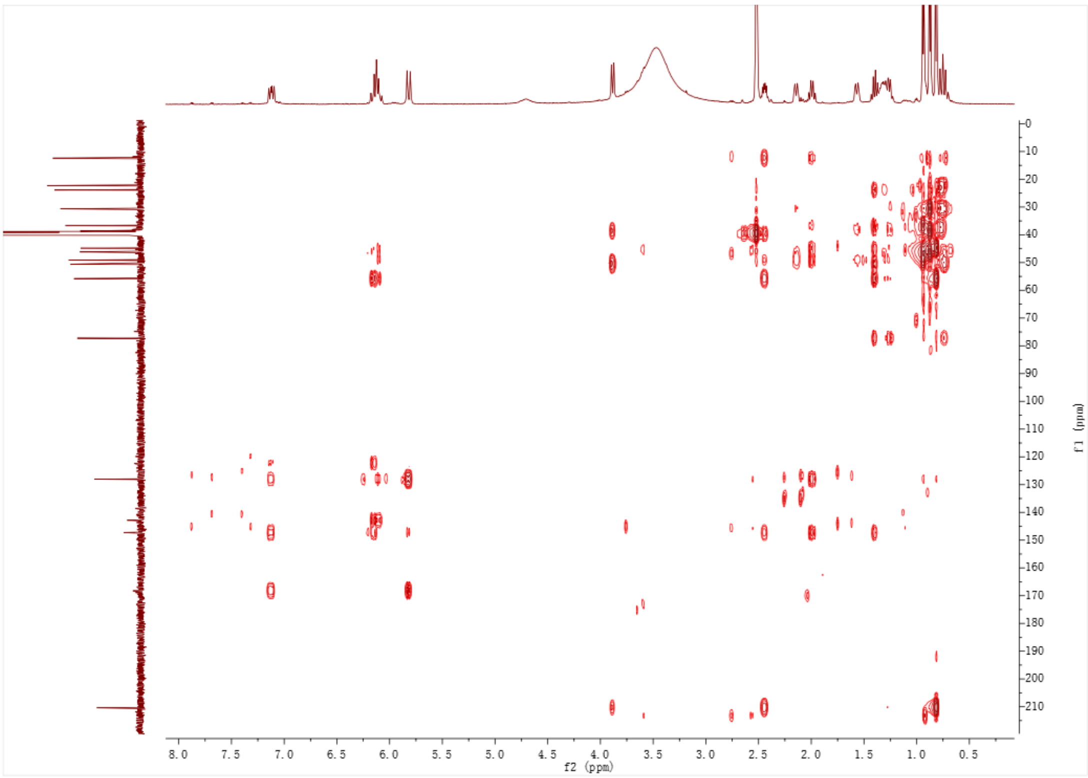

Figure S17 HMBC spectrum of compound **2** in DMSO-d_6

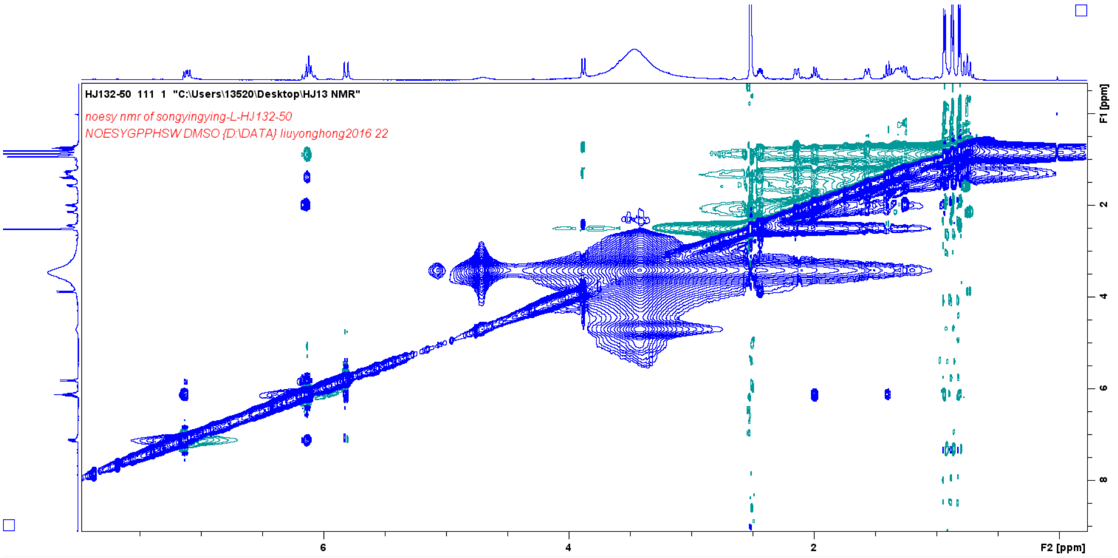

Figure S18 NOESY spectrum of compound **2** in DMSO-d_6

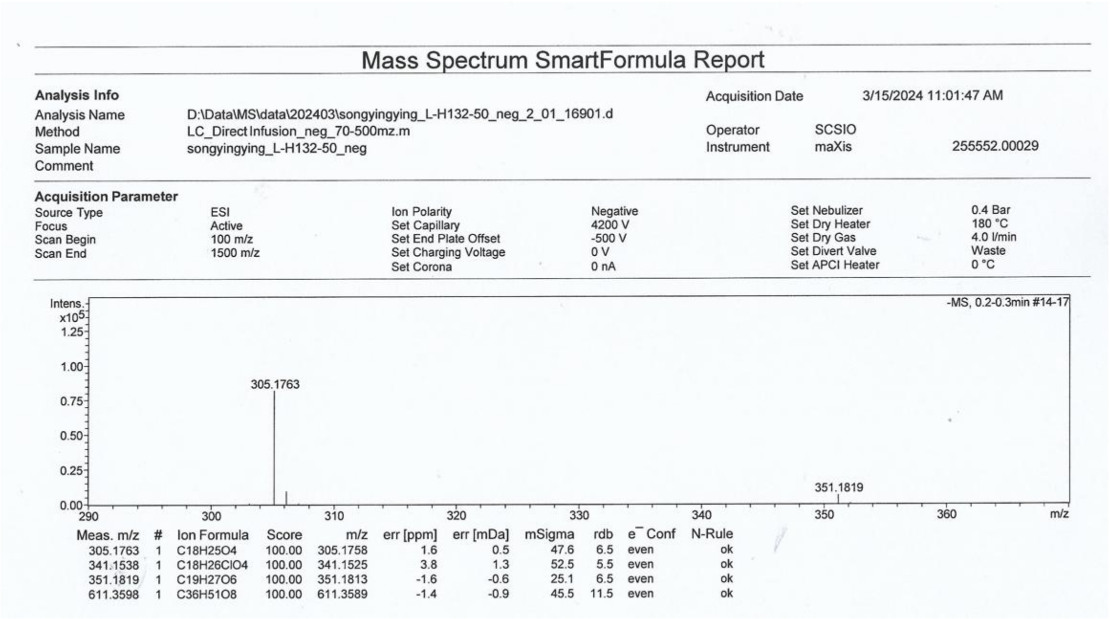

Mass Spectrum SmartFormula Report

Analysis Info

Analysis Name: D:\Data\MS\data\202403\songyingying_L-H132-50_neg_2_01_16901.d
Method: LC_Direct Infusion_neg_70-500mz.m
Sample Name: songyingying_L-H132-50_neg
Comment:

Acquisition Date: 3/15/2024 11:01:47 AM
Operator: SCSIO
Instrument: maXis 255552.00029

Acquisition Parameter

Source Type	ESI	Ion Polarity	Negative	Set Nebulizer	0.4 Bar
Focus	Active	Set Capillary	4200 V	Set Dry Heater	180 °C
Scan Begin	100 m/z	Set End Plate Offset	-500 V	Set Dry Gas	4.0 l/min
Scan End	1500 m/z	Set Charging Voltage	0 V	Set Divert Valve	Waste
		Set Corona	0 nA	Set APCI Heater	0 °C

Meas. m/z	#	Ion Formula	Score	m/z	err [ppm]	err [mDa]	mSigma	rdb	e⁻ Conf	N-Rule
305.1763	1	C18H25O4	100.00	305.1758	1.6	0.5	47.6	6.5	even	ok
341.1538	1	C18H26ClO4	100.00	341.1525	3.8	1.3	52.5	5.5	even	ok
351.1819	1	C19H27O6	100.00	351.1813	-1.6	-0.6	25.1	6.5	even	ok
611.3598	1	C36H51O8	100.00	611.3589	-1.4	-0.9	45.5	11.5	even	ok

Figure S19 HRESIMS spectrum of compound **2**

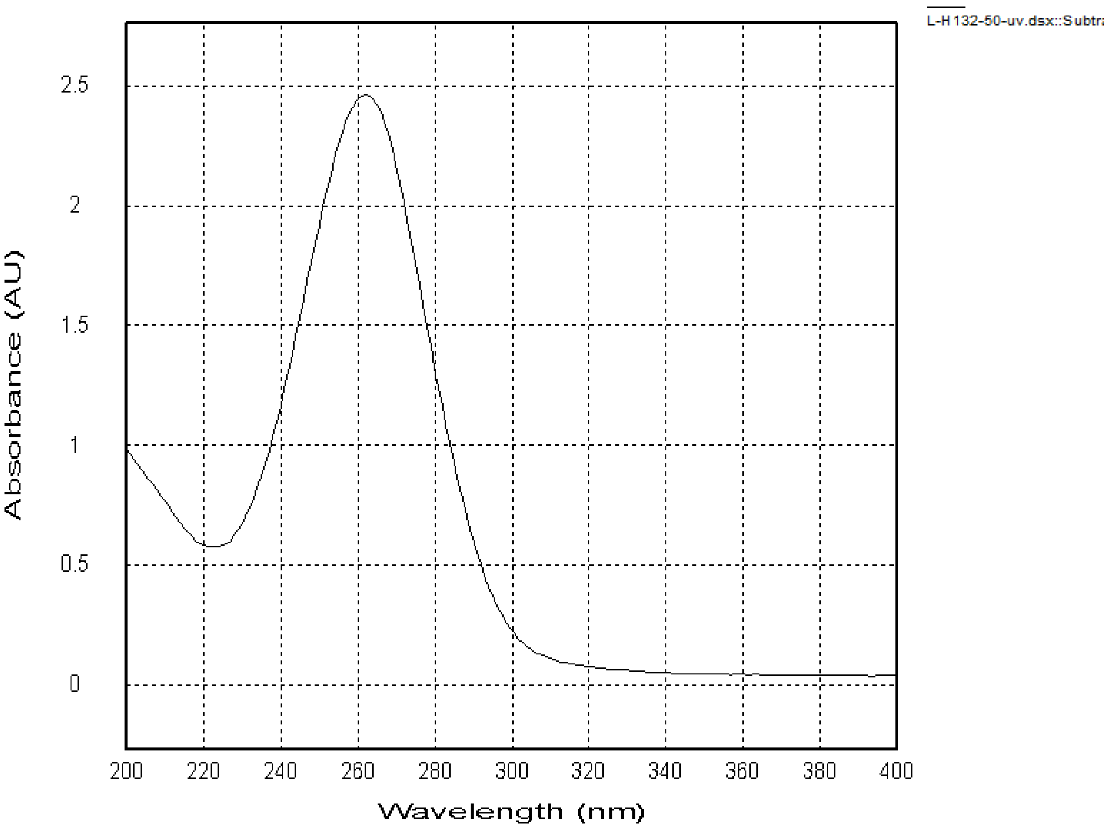

Figure S20 UV spectrum of **2**

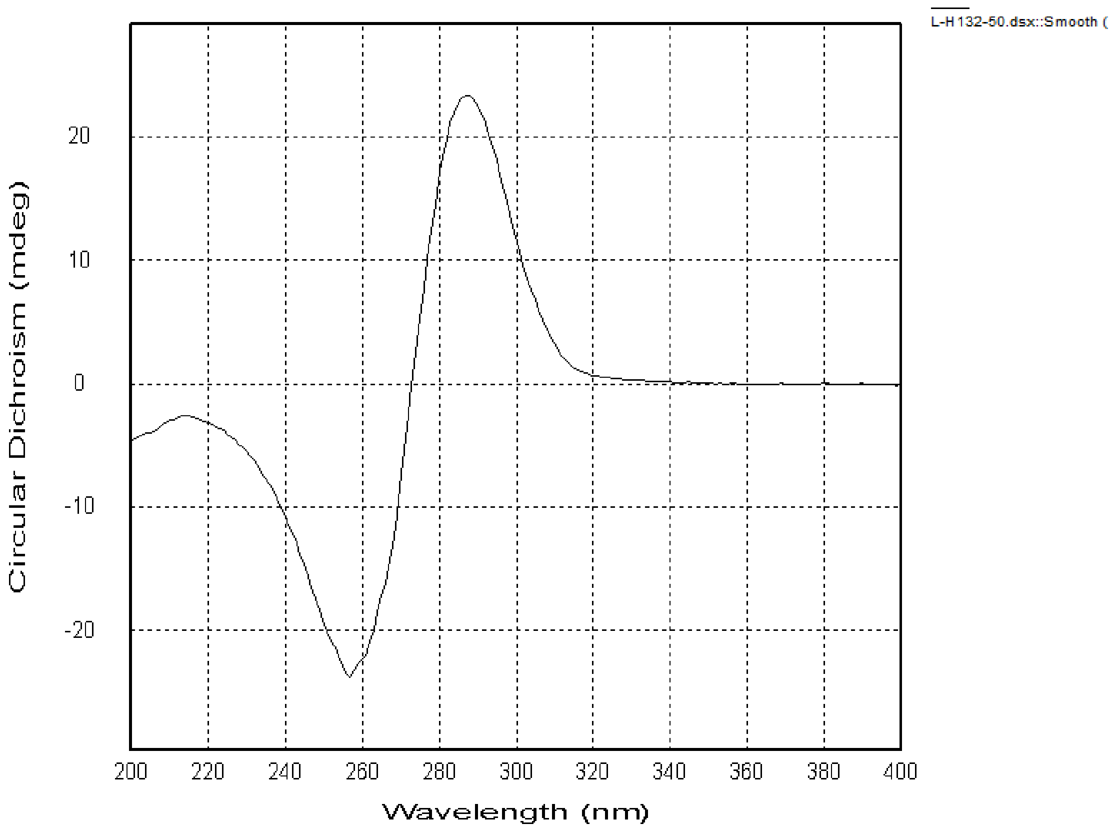

Figure S21 CD spectrum of **2**

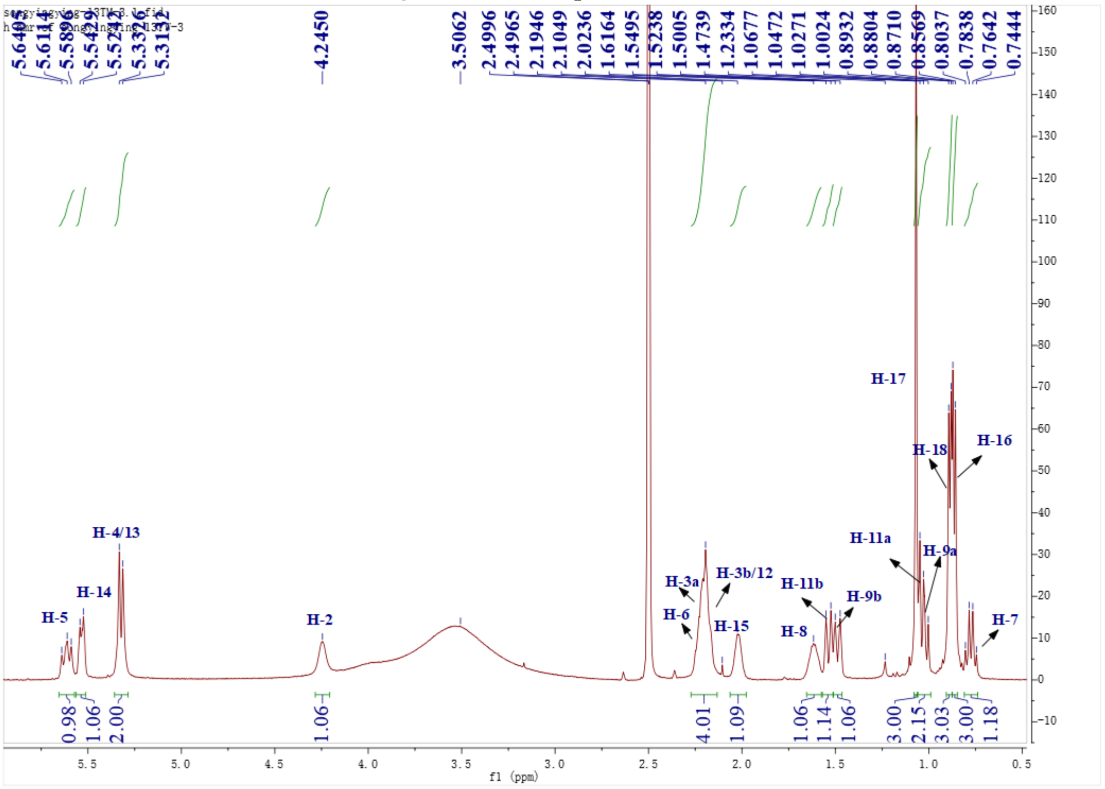

Figure S22 ^{1}H NMR spectrum of compound **3** in DMSO-d_6

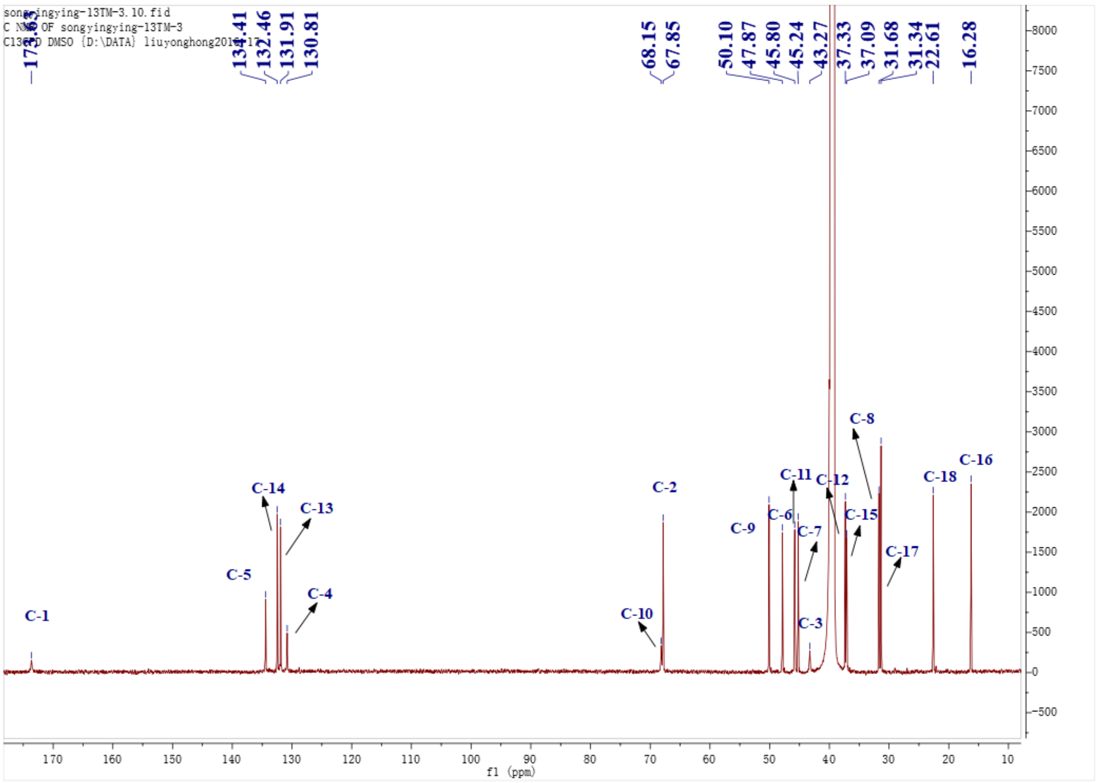

Figure S23 ^{13}C NMR spectrum of compound **3** in DMSO-d_6

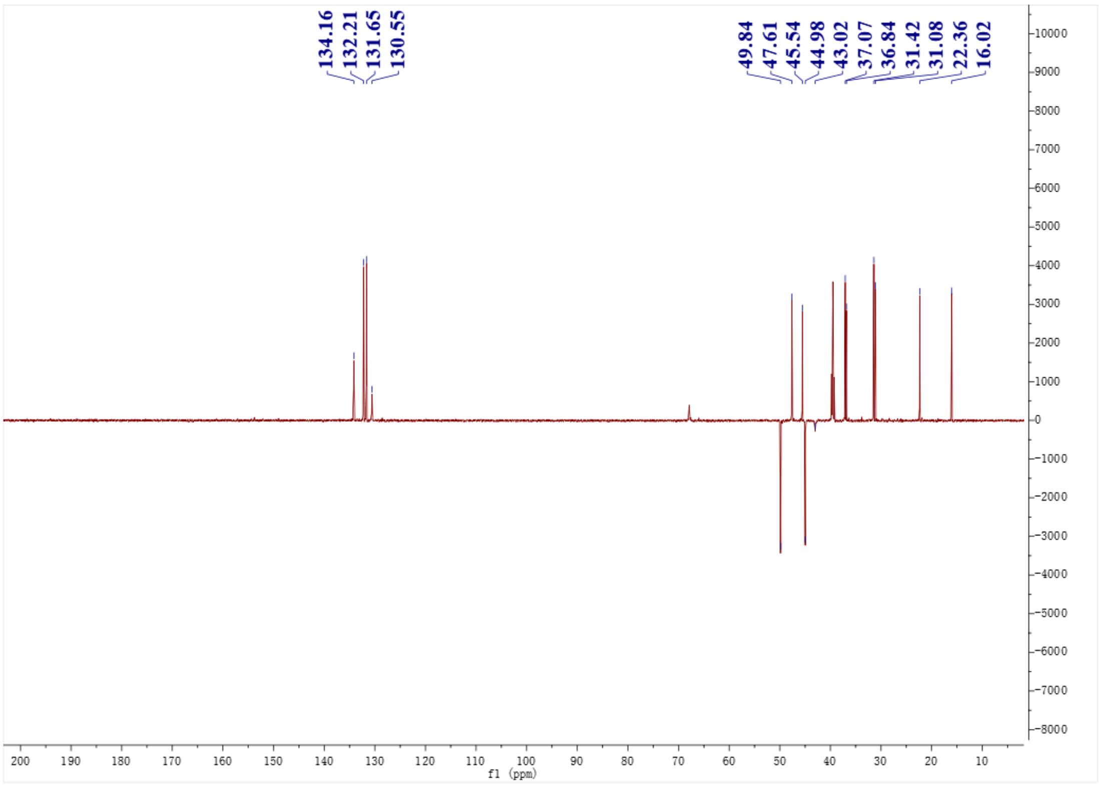

Figure S24 DEPT135 spectrum of compound **3** in DMSO-d_6

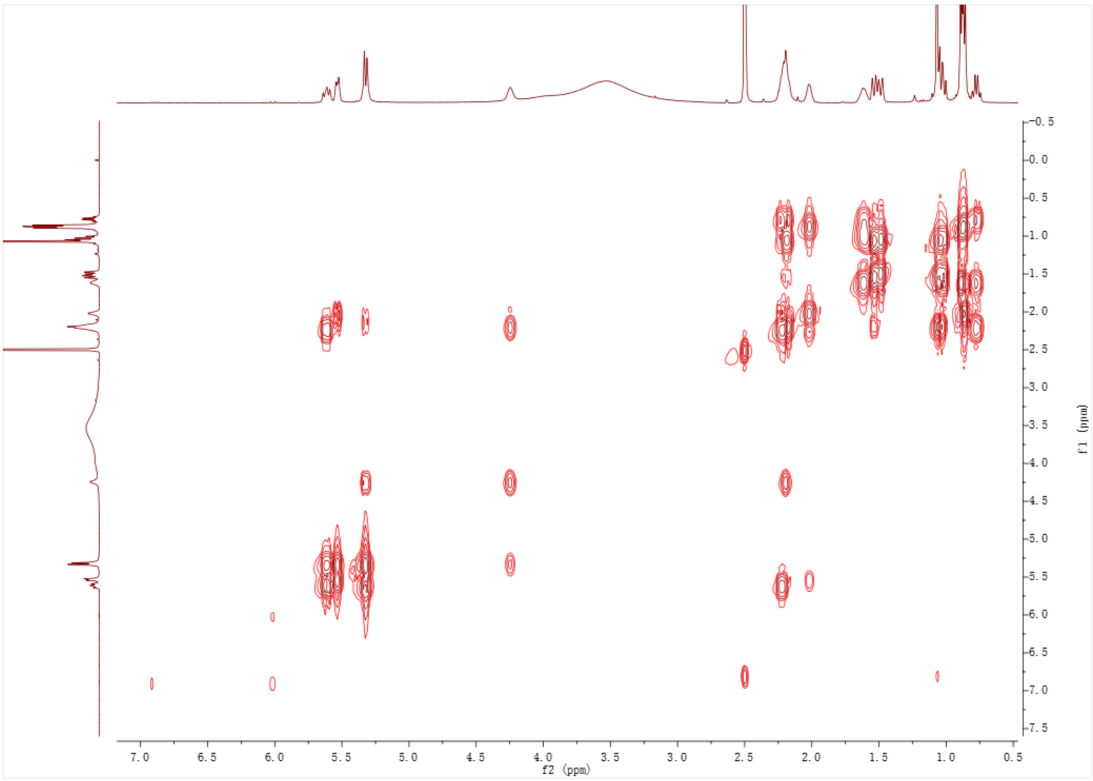

Figure S25 ^{1}H-^{1}H COSY spectrum of compound in DMSO-d_6

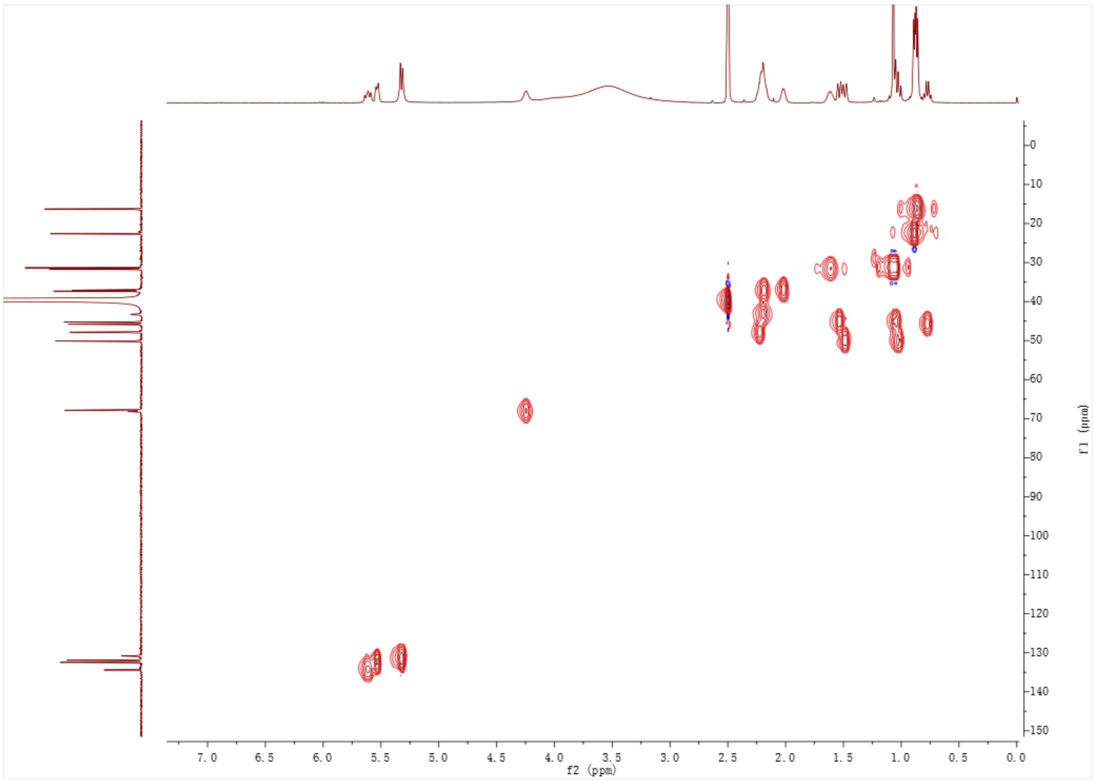

Figure S26 HSQC spectrum of compound **3** in DMSO-d_6

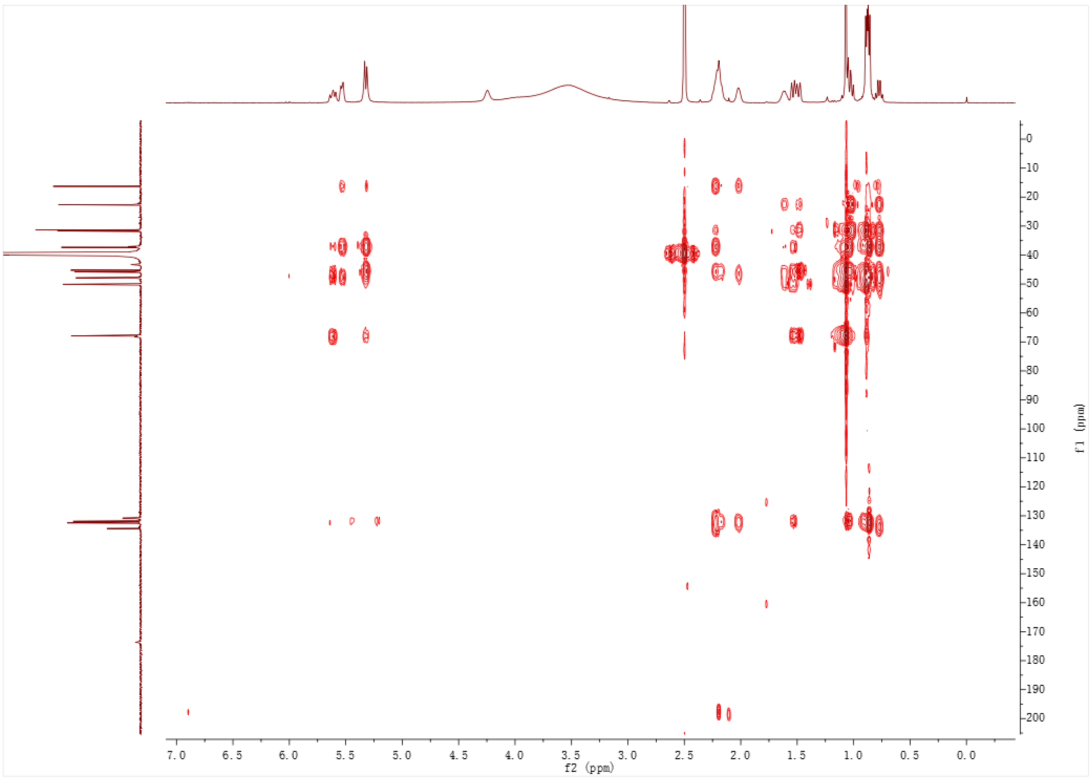

Figure S27 HMBC spectrum of compound **3** in DMSO-*d6*

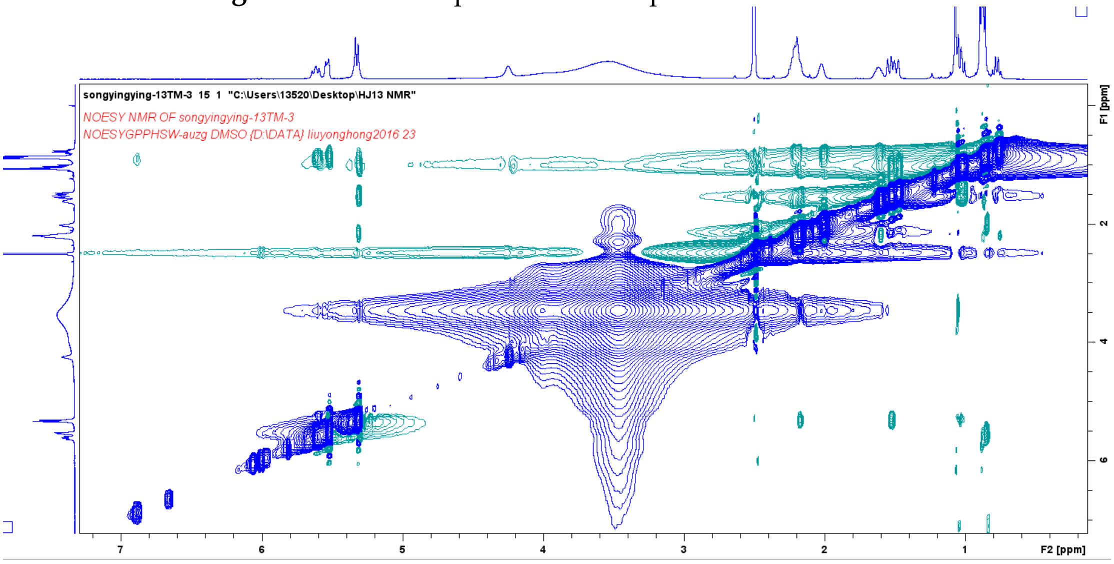

Figure S28 NOESY spectrum of compound **3** in DMSO-*d6*

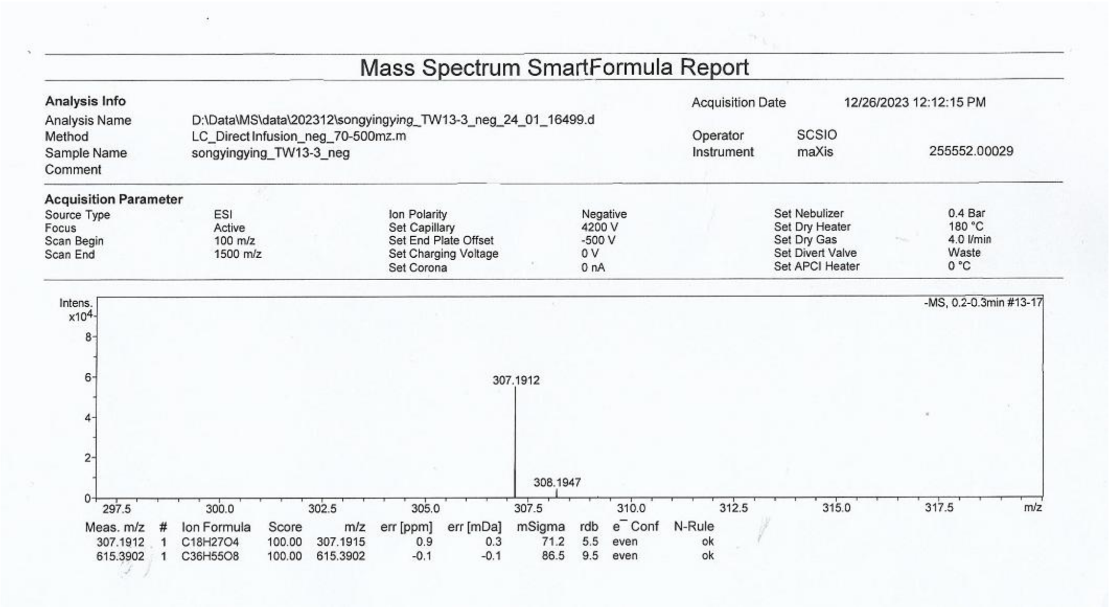

Figure S29 HRESIMS spectrum of compound **3**

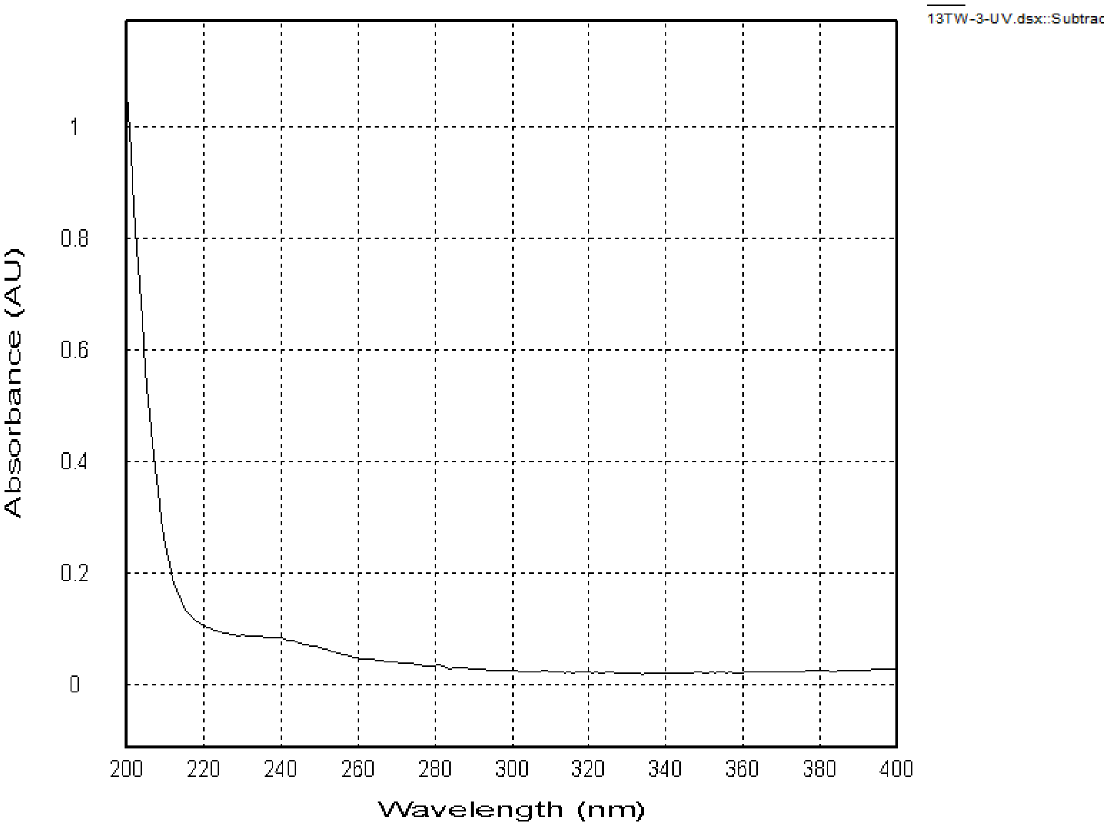

Figure S30 UV spectrum of **3**

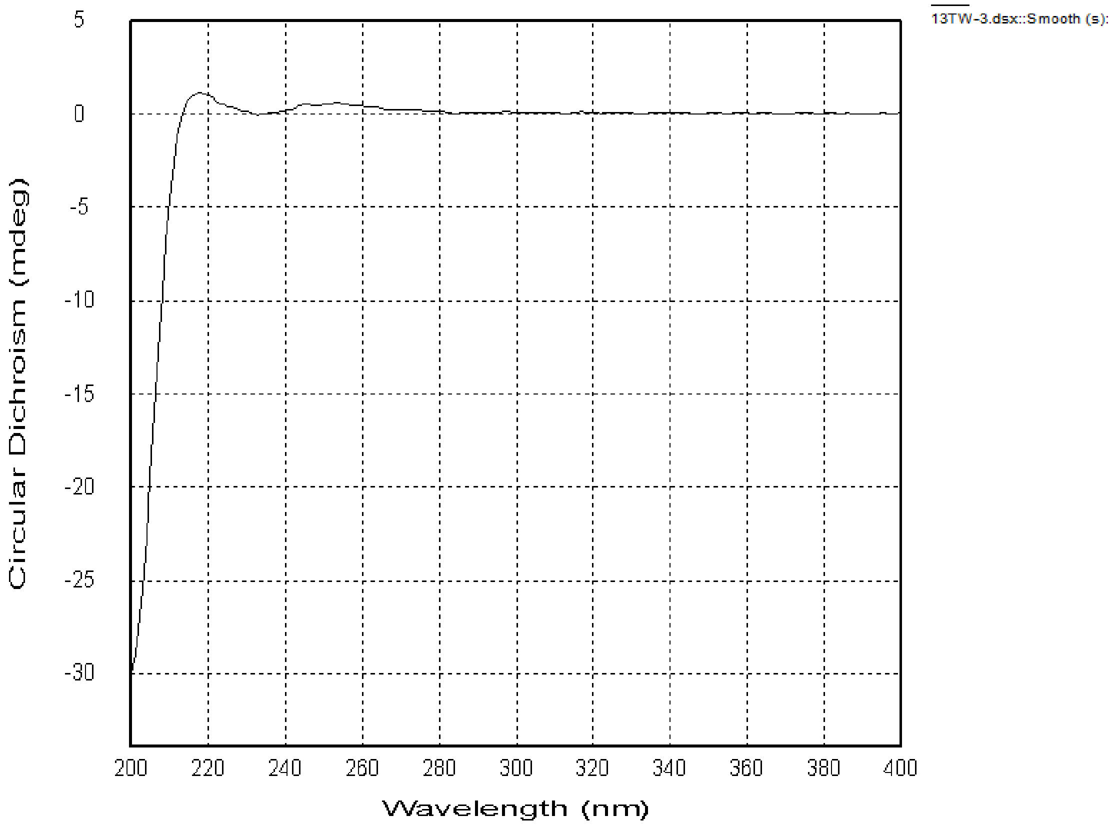

Figure S31 CD spectrum of **3**

		DP4+	98.19%	1.81%
Nuclei	sp2?	Experimental	Isomer 1	Isomer 2
C	x	173.6254	187.2	188.4
C	x	134.4122	160.1	160.5
C	x	131.9055	149.0	149.0
C	x	132.4649	146.6	146.7
C	x	130.8089	142.6	139.9
C		68.1543	84.4	84.2
C		67.8525	83.2	84.4
C		45.2355	57.7	57.7
C		47.8659	57.5	57.5
C		50.0972	54.2	55.0
C		45.8019	54.9	54.4
C		43.2731	52.37	49.99
C		37.0914	50.36	50.80
C		37.3251	47.97	47.99
C		31.6792	44.65	44.71
C		31.3382	41.69	41.65
C		16.2778	29.80	29.77
C		22.6144	28.33	28.31

Figure S32 DP4$^+$ analysis of calculated ^{13}C NMR data of **3**

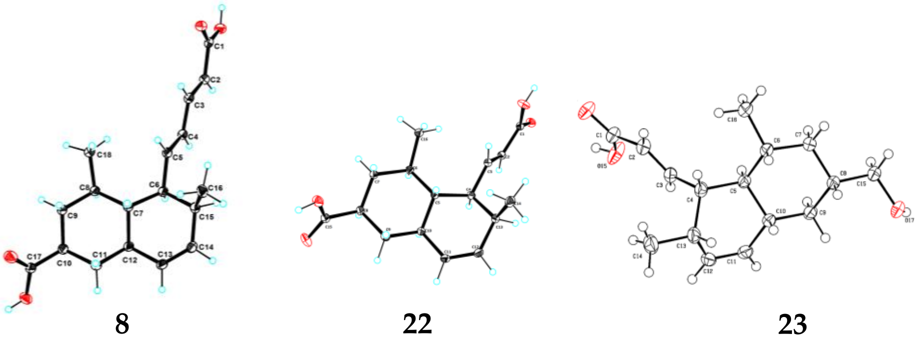

8 22 23

Figure S33 X-ray crystal structures of 8, 22 and 23

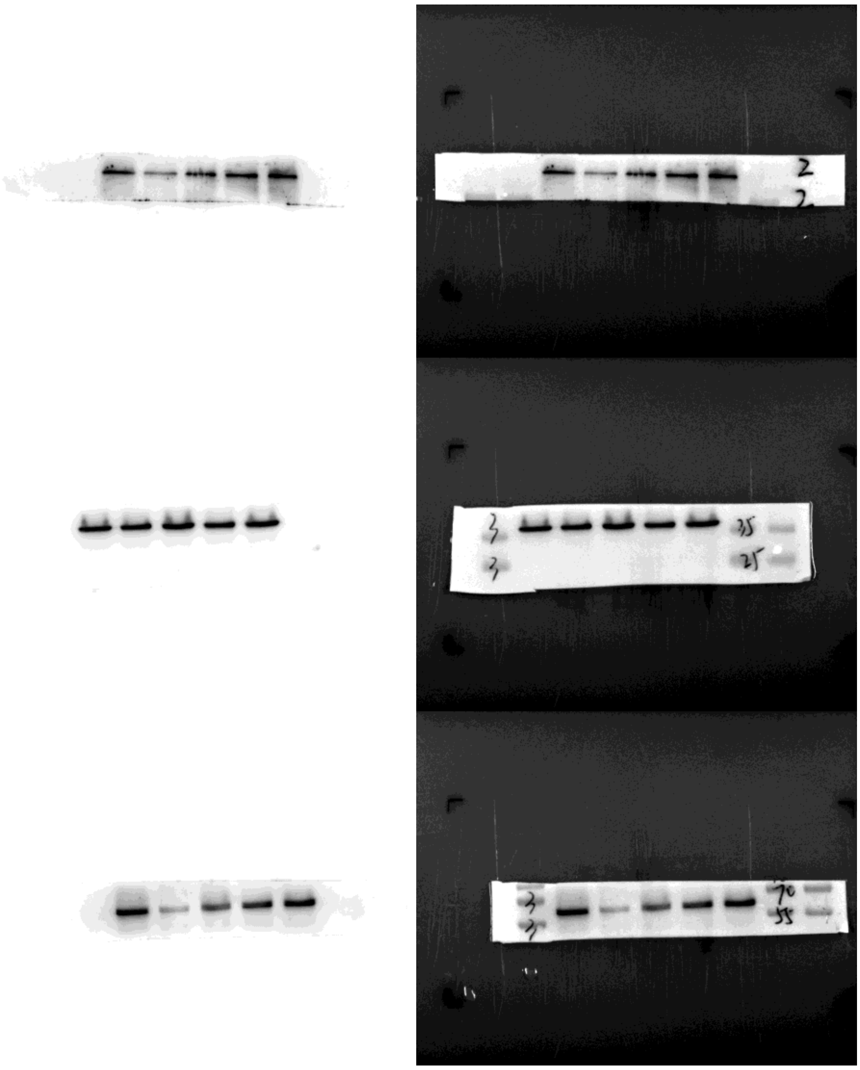

Figure S34 The original untreated photograph of the Western blot gel
